# High-dimensional analysis of T-cell profiling variations following belimumab treatment in systemic lupus erythematosus

**DOI:** 10.1136/lupus-2023-000976

**Published:** 2023-10-06

**Authors:** Shinji Maeda, Hiroya Hashimoto, Tomoyo Maeda, Shin-ya Tamechika, Shuntaro Isogai, Taio Naniwa, Akio Niimi

**Affiliations:** 1 Department of Respiratory Medicine, Allergy and Clinical Immunology, Nagoya City University Graduate School of Medical Sciences and Medical School, Nagoya, Japan; 2 Clinical Research Management Center, Nagoya City University Hospital, Nagoya, Japan

**Keywords:** T-lymphocytes, helper-inducer, lupus erythematosus, systemic, autoimmunity

## Abstract

**Objective:**

This study sought to elucidate the molecular impacts of belimumab (BEL) treatment on T-cell immune profiling in SLE.

**Methods:**

We used mass cytometry with 25 marker panels for T-cell immune profiling in peripheral blood T cells (CD3+) from 22 patients with BEL-treated SLE and 20 controls with non-BEL-treated SLE. An unsupervised machine-learning clustering, FlowSOM, was used to identify 39 T-cell clusters (TCLs; TCL01–TCL39). TCLs (% of CD3+) showing significant (p<0.05) associations with BEL treatment (BEL-TCL) were selected by a linear mixed-effects model for comparing groups of time-series data. Furthermore, we analysed the association between BEL treatment and variations in regulatory T-cell (Treg) phenotypes, and the ratio of other T-cell subsets to Treg as secondary analysis.

**Results:**

Clinical outcomes: BEL treatment was associated with a decrease in daily prednisolone use (coef=−0.1769, p=0.00074), and an increase in serum CH50 (coef=0.4653, p=0.003), C3 (coef=1.1047, p=0.00001) and C4 (coef=0.2990, p=0.00157) levels. Molecular effects: five distinct BEL-TCLs (TCL 04, 07, 11, 12 and 27) were identified. Among these, BEL-treated patients exhibited increased proportions in the Treg-like cluster TCL11 (coef=0.404, p=0.037) and two naïve TCLs (TCL04 and TCL07). TCL27 showed increased levels (coef=0.222, p=0.037) inversely correlating with baseline C3 levels. Secondary analyses revealed associations between BEL treatment and an increase in Tregs (coef=1.749, p=0.0044), elevated proportions of the fraction of Tregs with inhibitory function (fTregs, coef=0.7294, p=0.0178) and changes in peripheral helper T cells/fTreg (coef=−4.475, p=0.0319) and T helper 17/fTreg ratios (coef=−6.7868, p=0.0327). Additionally, BEL was linked to variations in T-cell immunoglobulin and mucin domain-containing protein-3 expression (coef=0.2422, p=0.039).

**Conclusions:**

The study suggests an association between BEL treatment and variations in T cells, particularly Tregs, in SLE pathologies involving various immune cells.

**WHAT IS ALREADY KNOWN ON THIS TOPIC**

- Belimumab improves immune pathogenesis by B cells; however, its effect on T cells remains unclear.

**WHAT THIS STUDY ADDS**

- High-dimensional analysis of peripheral blood T cells of patients with SLE using mass cytometry and FlowSOM machine learning identified five T-cell clusters and their phenotypes affected by belimumab, including one cluster of regulatory T cells (Tregs).
- Secondary analysis by manual gating confirmed an increase in Tregs and an improvement in the peripheral helper T cells/Tregs and T helper 17/Treg balance.

**HOW THIS STUDY MIGHT AFFECT RESEARCH, PRACTICE OR POLICY**

- The effect of B-cell therapeutics on T-cell immune tolerance will help explore the methodology of immunological remission in SLE pathology.

## Introduction

The prognosis of patients with SLE has not fully improved, and long-term prognosis is influenced by organ damage due to disease flares and side effects of therapeutic agents.^1 2^ Against this backdrop, belimumab (BEL), a B-lymphocyte-stimulating factor (BLyS)-specific inhibitor, emerges as a promising therapeutic strategy.^3–7^ Typically integrated into standard therapy, BEL acts to reduce disease activity by inhibiting excessive B-cell activation and proliferation in patients with SLE.^8–10^ BLyS overexpression in SLE has been associated with increased B-cell autoreactivity,^9 11–13^ suggesting that BEL can modify the autoreactive repertoire of peripheral blood B cells.^9^ Accordingly, BEL treatment improves inflammatory conditions caused by autoantibodies and immune complexes. Through this mechanism, BEL has been reported to successfully decrease disease activity, relapse rates and glucocorticoid use.^14^

Notably, the activation of autoreactive B cells produces high autoantibody titres.^15^ B cells are important in the aetiology of SLE, which involves various other immunocompetent cells.^15^ Within the realm of innate immunity, defective clearance of apoptotic cells and neutrophil extracellular traps by low-density granulocytes are critical for autoantigen exposure and autoantibody induction.^16–18^ Moreover, the sustained production of interferon (IFN)-α, predominantly by the plasmacytoid dendritic cells, triggers chronic inflammation.^19^ Among T cells, another immune cell involved in autoreactivity,^20^ the Th17 subset plays a significant role in the pathogenesis of SLE.^21–25^ Follicular helper T cells (Tfh) are essential for autoantibody production in lymphoid tissues and the generation of high-affinity autoantibodies.^26–28^ Peripheral helper T cells (Tph) also contribute significantly to the autoimmune response and autoantibody production in peripheral tissues.^29 30^ Regulatory T cells (Tregs) suppress these autoreactive inflammatory T cells and maintain immune tolerance.^31 32^ However, patients with SLE exhibit reduced interleukin-2 production by T cells, which is essential for Treg maintenance, suggesting that Tregs may be diminished or functionally impaired.^33^ This aberrant equilibrium between pathological T cells and Tregs is integral to the onset, activity and persistence of autoimmune diseases.^21 23 25^ B cell alterations during B-cell activating factor (BAFF) inhibition with BEL have garnered significant attention, with emphasis on both rapid and subsequent therapy-associated changes in B cell phenotypes.^34^ Furthermore, the intricate relationship between BLyS levels and the composition of both B and T cell compartments in patients with SLE undergoing BEL treatment has been expounded, elucidating the multifaceted effects of BEL on diverse immune cell subpopulations.^35^ Given these intricate dynamics in the immune response, particularly the roles of B and T cells in SLE, it becomes even more crucial to understand and manage the broader immunopathological landscape of the disease.

In order to effectively and rigorously manage SLE immunopathology and promote immunological remission, the overall imbalanced immune response should be rectified towards autoimmune tolerance. Consequently, it becomes imperative to investigate how current therapeutics might be associated with changes in their primary targets and in other pathological immune cells, and identify potential beneficial and detrimental shifts associated with treatments. Considering the undeniable role of T cells in SLE pathogenesis, the expression of the BAFF receptor on these cells,^36^ which is targeted by BEL, stands out as particularly significant. BAFF plays a pivotal role in T cell activation and profoundly influences the differentiation of T cell subsets.^37^ These insights further underscore the potential therapeutic implications of BEL beyond its primary targets.

The primary objective of this study was to investigate the potential associations between BEL administration and alterations in T cell subpopulation distribution, using mass cytometry to analyse peripheral blood T cells from patients with SLE. Recent studies underscore the significance of T cell roles in SLE pathogenesis. Our findings aim to provide valuable insights into the effects of BEL treatment on T cell dynamics, which could potentially influence future therapeutic strategies.

## Materials and methods

### Participants

This study enrolled 22 outpatients with SLE who had started receiving BEL (BEL group, BEL-G) and 20 patients with SLE who did not receive BEL (control group, CON-G) at Nagoya City University Hospital (online supplemental figure 1). SLE was confirmed based on the American College of Rheumatology criteria.^38^ BEL use was determined by consensus between the treating physician and patient during routine medical care based on disease activity, symptoms, prescribed medications and treatment history. In BEL-G, BEL (10 mg/kg) was intravenously administered on days 0, 14 and 28 and monthly thereafter for≥12 months. Patients were included if their glucocorticoid and immunosuppressant doses were not changed during 6 months before starting BEL treatment.

10.1136/lupus-2023-000976.supp1 Supplementary data

### Clinical and laboratory assessment

The following clinical variables were obtained from medical records: age; sex; disease duration; clinical manifestations; Physician Global Assessment (0–3); use of corticosteroids, immunosuppressive drugs (azathioprine, ciclosporin, tacrolimus, mizoribine, mycophenolate mofetil, methotrexate and hydroxychloroquine) and non-steroidal anti-inflammatory drugs; complete blood count; antidouble-stranded DNA antibody (ELISA: BEL-G, n=20; CON-G, n=12) or anti-DNA antibody (radioimmunoassay (RIA), BEL-G, n=21; CON-G, n=12) titre; serum complement C3 and C4 levels; serum complement haemolytic activity (CH50); presence of antiphospholipid antibodies and urine protein-to-creatinine ratio (g/g Cr). The SLE-Disease Activity Index-2000 (SLEDAI-2K), a validated clinimetric index, was used to assess disease activity.^39^ These clinical variables were evaluated in BEL-G before (0M), 3 months (3M) and 12 months (12M) after BEL treatment and in CON-G (non-BEL) at the initial reference blood collection (0M) and 12 months later (12M). Clinical manifestations of SLE (articular, cutaneous, neuropsychiatric, renal and serositis) at baseline were considered symptomatic using the SLEDAI-2K score,^39^ if any of the following were present: articular: arthritis; cutaneous: rash and alopecia; neuropsychiatric: seizures, psychosis and organic brain syndrome; renal: urinary casts, haematuria, proteinuria and pyuria and serositis: pleurisy and pericarditis.

### Cell surface staining and mass cytometric analysis

Peripheral blood (10 mL) was drawn from outpatients with SLE in heparinised collection tubes. Peripheral blood mononuclear cells (PBMCs) were isolated from the blood samples by density gradient centrifugation using Leucosep (Greiner Bio-One, Kremsmuenster, Austria) with Ficoll-Paque Plus (Cytiva, Tokyo, Japan), L-glutamine and phenol red (FUJIFILM). PBMCs were then cryopreserved in a deep freezer at −80°C using Cell Banker 1 plus (Takara Bio, Japan) as the base medium. The cryopreservation period did not exceed 18 months. To minimise cell damage during freezing, we used Corning CoolCell LX Cell Freezing Containers (Corning, Corning, New York, USA). Cryopreserved PBMCs were lysed in an incubator at 37°C and washed with Maxpar Cell Staining Buffer (Fluidigm, South San Francisco, California, USA). To identify dead cells, PBMCs were incubated with 0.1 M of cisplatin using Cell-ID Cisplatin-198Pt (Fluidigm). To avoid non-specific epitope binding, cells were blocked with Human Tru Stain FcX (BioLegend, San Diego, California, USA).

Each sample of 1 million cells was barcoded using the CELL-ID 20 plex PD Barcording Kit (Fluidigm) per the kit protocol. Overall, 20 barcoded samples were pooled into one sample for analysis and subsequent staining. Two common samples were included in all combined samples; variations in data due to different analysis dates could be adjusted and controlled in each analysis sample.

A cocktail of 25 metal isotope-labelled monoclonal antibodies (Fluidigm) was prepared for cell surface staining. The 25 target cell surface antigens of the monoclonal antibodies were CD3, CD4, CD8, CD45RO, CD45RA, C-C motif chemokine receptor (CCR)7, human leucocyte antigen-DR isotype (HLA-DR), CD38, CD25, CD127, CXCR3, CCR5, CCR4, CCR6, CD161, CXCR5, programmed death-1 (PD-1), CD28, cytotoxic T-lymphocyte-associated antigen-4 (CTLA-4), lymphocyte-activation gene 3 (LAG-3), inducible T-cell costimulator (ICOS), tumour necrosis factor receptor superfamily member 9 (4-1BB), OX40, Fas and T-cell immunoglobulin and mucin domain containing protein-3 (TIM-3) (online supplemental figure 1). Details of the metal isotope-labelled monoclonal antibodies used in this study are provided in online supplemental table 1.

10.1136/lupus-2023-000976.supp8 Supplementary data

Furthermore, each sample for analysis was stained with the antibody cocktail for 1 hour at 4°C. After centrifugation, cells were washed and fixed in 1.6% formaldehyde using 16% formaldehyde solution (w/v) (Thermo Scientific, Massachusetts, USA) diluted in Maxpar PBS (Fluidigm). These samples were sent by refrigerated mail to St. Luke’s SRL Advanced Clinical Research Center (Tokyo Japan) for contract analysis. Cell samples were washed and incubated overnight at 4°C in Maxpar Fix and Perm Buffer (Fluidigm) with 125 nM iridium intercalator (Cell-ID Intercalator-Ir 125 μM, Fluidigm). Each cell sample was then washed with Milli-Q water and filtered through a 35 μm nylon mesh. Moreover, EQ Four Element Calibration Beads (Fluidigm) were added per the instrument protocol. The samples were analysed using Helios mass cytometer and CyTOF System (Fluidigm). Cytometric data from System were the CyTOF exported in FCS 3.0 file format.

### Analysis of peripheral T-cell subsets by mass cytometry

Using cytometric data, we analysed the percentage (%) of each T-cell subset among CD3+ T cells in the peripheral blood (online supplemental figures 1; 2). The T-cell subsets were as follows: Th1, CD3+CD4+CD8−CD45RO+CXCR3+CCR4−CCR6−; Th2, CD3+CD4+CD8−CD45RO+CCR4+CXCR3−CCR6−; Th17, CD3+CD4+CD8−CD45RO+CCR6+CXCR3−; Th17.1, CD3+CD4+CD8−CD45RO+CCR6+CD161+CXCR3+CCR4−; Tfh, CD3+CD4+CD8−CD45RO+PD-1high+CXCR5+ICOS+; Tph, CD3+CD4+CD8−CD45RO+PD-1high+CXCR5−ICOS+; Treg, CD3+CD4+CD25+CD127−; Treg fraction I^40^ (naïve Treg), CD45RA+CD25low+Treg; Treg fraction II^40^ (effector Treg), CD25high+CD45RA− Tregs; Treg fraction III,^40^ CD25low+CD45RA− Tregs; central memory (CM) T cells, CCR7+CD45RA−; effector memory (EM) T cells, CCR7−CD45RA−; effector T cells, CCR7−CD45RA+ and naïve T cells, CCR7+CD45RA+. Analysis was performed using FlowJo V.10.8.1 (TreeStar, Ashland, Oregon, USA). Expression levels of surface molecules on Tregs were analysed using FlowJo.

10.1136/lupus-2023-000976.supp2 Supplementary data

### Clustering of CD3+ T cells using unsupervised machine-learning FlowSOM

Unsupervised clustering was performed using FlowSOM to target the CD3+ gated cell population of the CyTOF concatenated data in 42 participants. Gating steps performed before CD3+ T cell gating were the removal of residual beads with 140Ce_beads, exclusion of doublet cells with Event_length and 191Ir and exclusion of dead cells with 191Ir and 198Pt. For the clustering process, we employed the FlowSOM algorithm,^41^ integrated within the Bioconductor’s CATALYST R package (V.1.16.0).^42 43^ Recognising the vast dynamic range of signals inherent to CyTOF data, raw expression values were subjected to an arcsinh transformation, using a cofactor of 5 (online supplemental figure 1). This transformation procedure addresses the wide-ranging dynamism inherent to the CyTOF data, facilitating a more refined clustering. For pooled samples, FlowSOM method in the CATALYST R package was used. FlowSOM clustering was performed using all 25 T-cell markers from the panel on a 10×10 grid with a maximum of K=39 clusters predefined in ConsensusClusterPlus. Then, 39 T-cell clusters (TCLs; TCL01–TCL39) were identified. Their percentages were calculated using the R and CATALYST R packages. High-dimensional concatenated data of 25 T-cell markers were visualised in a two-dimensional plot using t-distributed stochastic neighbour embedding (t-SNE).

### Statistical analysis

Regarding patient background, differences between BEL-G and CON-G were evaluated using the Mann-Whitney U test and Fisher’s exact test for continuous and categorical variables, respectively. A linear mixed-effects model was used to examine changes in clinical indicators, TCLs, T-cell subsets and levels of surface molecules on Tregs by BEL intervention. Time, BEL intervention (group) and interaction between time and group were considered fixed effects, with the sample as a random effect. Coefficients of interaction between group and time and their p values were reported. All calculated p values were two-sided, with p<0.05 considered significant. All statistical analyses were performed using R software V.4.1.2 (R Development Core Team, Vienna, Austria) and EZR V.1.54 (Saitama Medical Center, Jichi Medical University, Saitama, Japan).^44^ R software packages ggplot2 (V.3.4.0)^45^ and lmerTest (V.3.1-3)^46^ were used for statistical processing and graph and table creation.

### Patient and public involvement

Patients and/or the public were not involved in the design, or conduct, or reporting, or dissemination plans of this research.

## Results

### Baseline characteristics of patients with SLE

The study included 42 patients with SLE (BEL-G: n=22; CON-G: n=20) (table 1). The disease activity index SLEDAI-2K (BEL-G, 8.00 (median); CON-G, 5.50; p=0.017) and Physician Global Assessment score (BEL-G, 0.90 (median); CON-G, 0.55; p=0.003) were significantly higher in BEL-G than in CON-G. Among clinical symptoms, skin symptoms (59.1% and 15% in BEL-G and CON-G, respectively, p=0.005) were significantly more common in BEL-G. Regarding medication, glucocorticoid dosage (BEL-G, 7.5 mg/day (median); CON-G, 4.5 mg/day; p=0.00051) and mycophenolic acid use (BEL-G, 27.3%; CON-G, 0%; p=0.02) were significantly higher in BEL-G. Immunological findings revealed significantly higher levels of antidouble-stranded DNA antibodies in BEL-G (BEL-G, 17.5 IU/mL (median); CON-G, 10.0 IU/mL, p=0.02), whereas serum complement titres, CH50 (BEL-G, 39.8 µ/mL (median); CON-G, 50. 35 IU/mL, p=0.02) and C4 (BEL-G, 11.5 mg/dL (median); CON-G, 14.0 mg/dL, p=0.02), were significantly lower in BEL-G, indicating a higher serological activity in BEL-G before BEL treatment.

**Table 1:** Clinical characteristics of patients with SLE

	CON-G, n=20	BEL-G, n=22	P value
Age, year	51.49 (38.55, 55.14)	47.52 (33.48, 54.92)	0.378
Sex, male/female (%)	2 (10.0)/18 (90.0)	0 (0.0)/22 (100.0)	0.221
Disease duration, year	15.39 (9.84, 30.50)	17.09 (7.87, 24.12)	0.669
Clinical manifestations of SLE			
Articular (%)	3 (15.0)	6 (27.3)	0.46
Cutaneous (%)	3 (15.0)	13 (59.1)	0.005*
Neuropsychiatric symptoms (%)	1 (5.0)	2 (9.1)	1
Renal (%)	2 (10.0)	8 (36.4)	0.071
Serositis (%)	0 (0)	0 (0)	n.a.
SLEDAI-2K	5.50 (3.50, 6.00)	8.00 (6.00, 11.75)	0.017*
Physician Global Assessment (0–3 points)	0.55 (0.30, 0.83)	0.90 (0.72, 1.17)	0.003*
Concomitant glucocorticoids (%)	20 (100.0)	22 (100.0)	n.a.
Daily dose of prednisolone equivalent, mg	4.50 (3.12, 5.25)	7.50 (5.00, 9.75)	0.00051*
Concomitant immunosuppressive and immunomodulatory agents (%)			
Azathioprine (%)	6 (30.0)	7 (31.8)	1
Ciclosporin (%)	1 (5.0)	3 (13.6)	0.608
Tacrolimus (%)	7 (35.0)	13 (59.1)	0.137
Mizoribine (%)	4 (20.0)	3 (13.6)	0.691
Mycophenolates (%)	0 (0.0)	6 (27.3)	0.022*
Methotrexate (%)	1 (5.0)	0 (0.0)	0.476
Hydroxychloroquine (%)	3 (15.0)	2 (9.1)	0.656
Number of concomitant immunosuppressive and immunomodulatory agents (%)			
0	4 (20.0)	1 (4.5)	0.288
1	11 (55.0)	10 (45.5)	
2	4 (20.0)	9 (40.9)	
3	1 (5.0)	2 (9.1)	
Non-steroidal anti-inflammatory drugs (%)	0 (0.0)	1 (4.5)	1
Leucocytes (×10^6^/mm^3^)	4.75 (3.75, 6.93)	4.65 (3.95, 6.57)	0.791
Lymphocytes (×10^6^/mm^3^)	1.13 (0.72, 1.45)	0.81 (0.39, 1.27)	0.128
Haemoglobin (g/L)	128.0 (114.3, 136.5)	116.0 (101.5, 126.7)	0.022*
Platelets (×10^6^/mm^3^)	232.50 (202.25, 284.25)	222.00 (173.25, 252.75)	0.273
Antidouble-stranded DNA IgG antibody<ELISA>, IU/mL, ≤12	10.00 (10.00, 10.25)	17.50 (10.00, 42.00)	0.01*
Anti-DNA antibody<RIA>, IU/mL, ≤6	2.00 (2.00, 6.00)	13.00 (3.80, 21.00)	0.024*
C3 (mg/dL, 73–138)	69.00 (63.50, 92.00)	62.00 (52.25, 79.50)	0.016*
C4 (mg/dL, 11–31)	14.00 (10.50, 21.00)	11.50 (8.00, 14.75)	0.094
CH50 (U/mL, 32–48)	50.35 (36.78, 58.17)	39.80 (30.12, 51.60)	0.04*
Antiphospholipid antibody positive (%)	2 (10.0)	8 (36.4)	0.071
Urine protein-to-creatinine ratio, g/g·Cr, <0.15	0.00 (0.00, 0.11)	0.10 (0.01, 0.26)	0.032*

*Statistical significance level of p < 0.05.

BEL-G, belimumab group; CON-G, control group; RIA, radioimmunoassay; SLEDAI-2K, SLE-Disease Activity Index 2000.

### Changes in clinical indicators with BEL treatment

**Figure 1:**
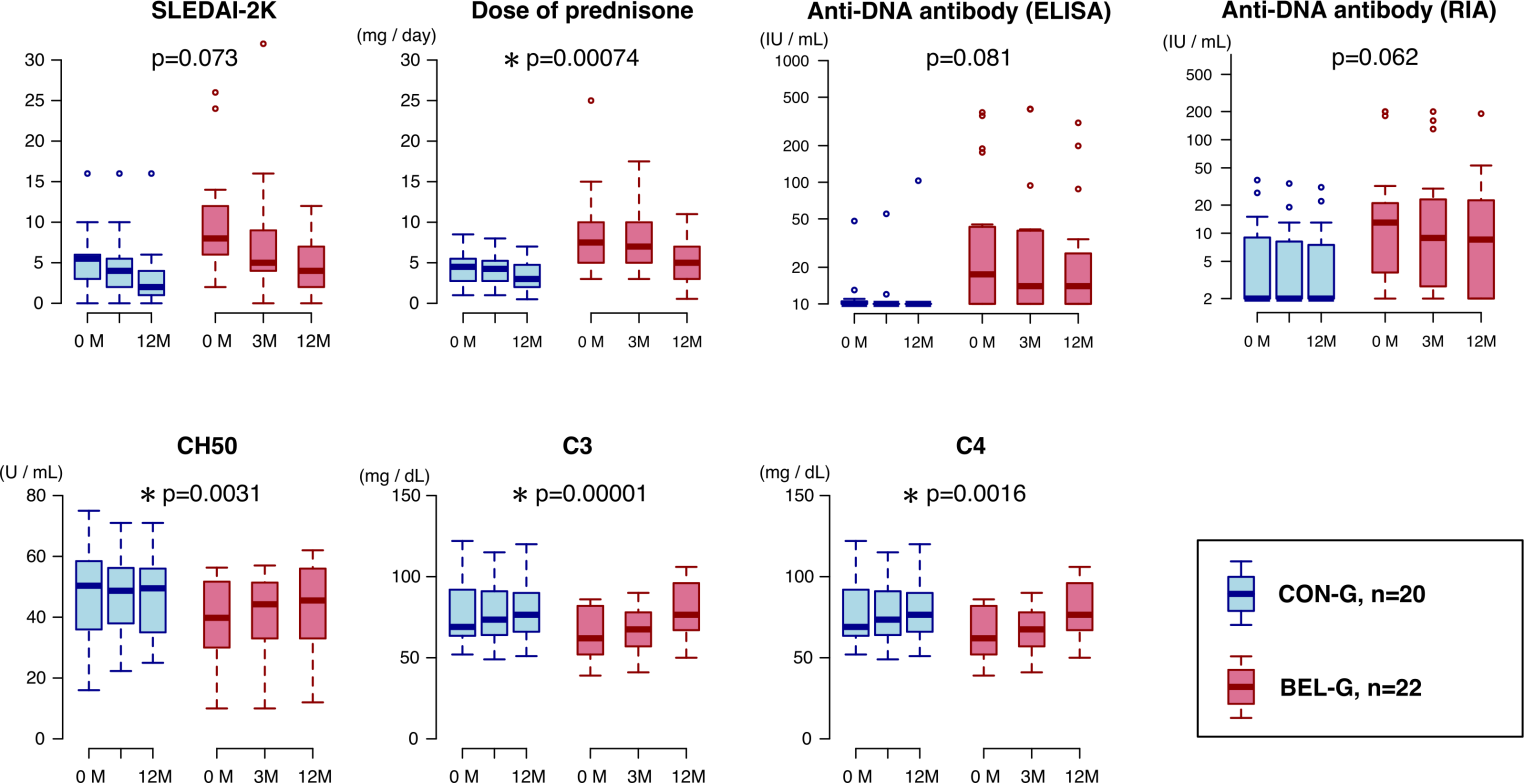
Changes in the clinical parameters by belimumab treatment. The figure illustrates changes in SLE-Disease Activity Index-2000 (SLEDAI-2K), oral steroid dose (prednisolone equivalent daily dose), antidouble-stranded DNA IgG antibody (assessed by ELISA), anti-DNA antibody (assessed by radioimmunoassay) and complement titres (CH50, C3 and C4) in both belimumab group (BEL-G) and control group (CON-G) using box-and-whisker plots. Comparisons were made between the two groups, with p values calculated using a linear mixed-effects model. A p value <0.05 was considered significant and is denoted by an asterisk.

Changes in clinical indices in BEL-G are shown in online supplemental table 2. The changes in each clinical index at 0M, 3M and 12M after BEL treatment were compared with the changes at 0M and 12M in CON-G using a mixed-effects model. Daily glucocorticoid dose (prednisolone equivalent) decreased significantly (p=0.00074), whereas the serum complement titres CH50 (p=0.0039), C3 (p=0.00001) and C4 (p=0.00157) increased significantly. SLEDAI-2K (p=0.072) and antidouble-stranded DNA antibodies (p=0.08) tended to improve but not significantly (figure 1).

10.1136/lupus-2023-000976.supp9 Supplementary data

### Baseline T-cell characteristics

First, the peripheral blood T-cell subset of each patient was analysed by manual gating using data measured by CyTOF (online supplemental figure 2). Differences in baseline peripheral blood T-cell subsets are shown in online supplemental table 3. Regarding the percentage of each CD4 helper T-cell subset in CD4+ T cells, Th17 (p=0.039) and Th2 (p=0.027) were significantly higher in BEL-G. We analysed Tregs in fractions (online supplemental figure 2): fraction I (CD25 low+CD45RA+), which is naïve and has an inhibitory function; fraction II (CD25 high+CD45RA−), an effector that has a high inhibitory function and fraction III (CD25low+CD45RA−), which has no inhibitory function. Fractions I and II were combined to form a functional Treg (fTreg).^40^ Activated (HLA-DR+CD38+) fractions of Tregs were also analysed. But there were no differences in Treg at baseline. Regarding the ratio of each Th subset to fTreg, Th2/fTreg (p=0.004), Th17/fTreg (p=0.009) and Tph/fTreg (p=0.008) were significantly higher in BEL-G. The percentages of activated T cells (CD38+HLA-DR+), CM, effector, EM and naïve fractions were also compared between CD4+ and CD8 T cells. Notably, the percentage of activation in CD4+ (p=0.015) and CD8+ (p=0.048) T cells was significantly higher in BLE-G than in CON-G. Conversely, the percentage of CD4+ T cells in the effector fraction was significantly higher in CON-G than in BEL-G. At baseline, T-cell activation and percentage of pathological T cells (Th17/fTreg and Tph/fTreg) were higher in BEL-G than in CON-G.

10.1136/lupus-2023-000976.supp10 Supplementary data

### T-cell clustering analysis using self-organising maps (FlowSOM)

**Figure 2:**
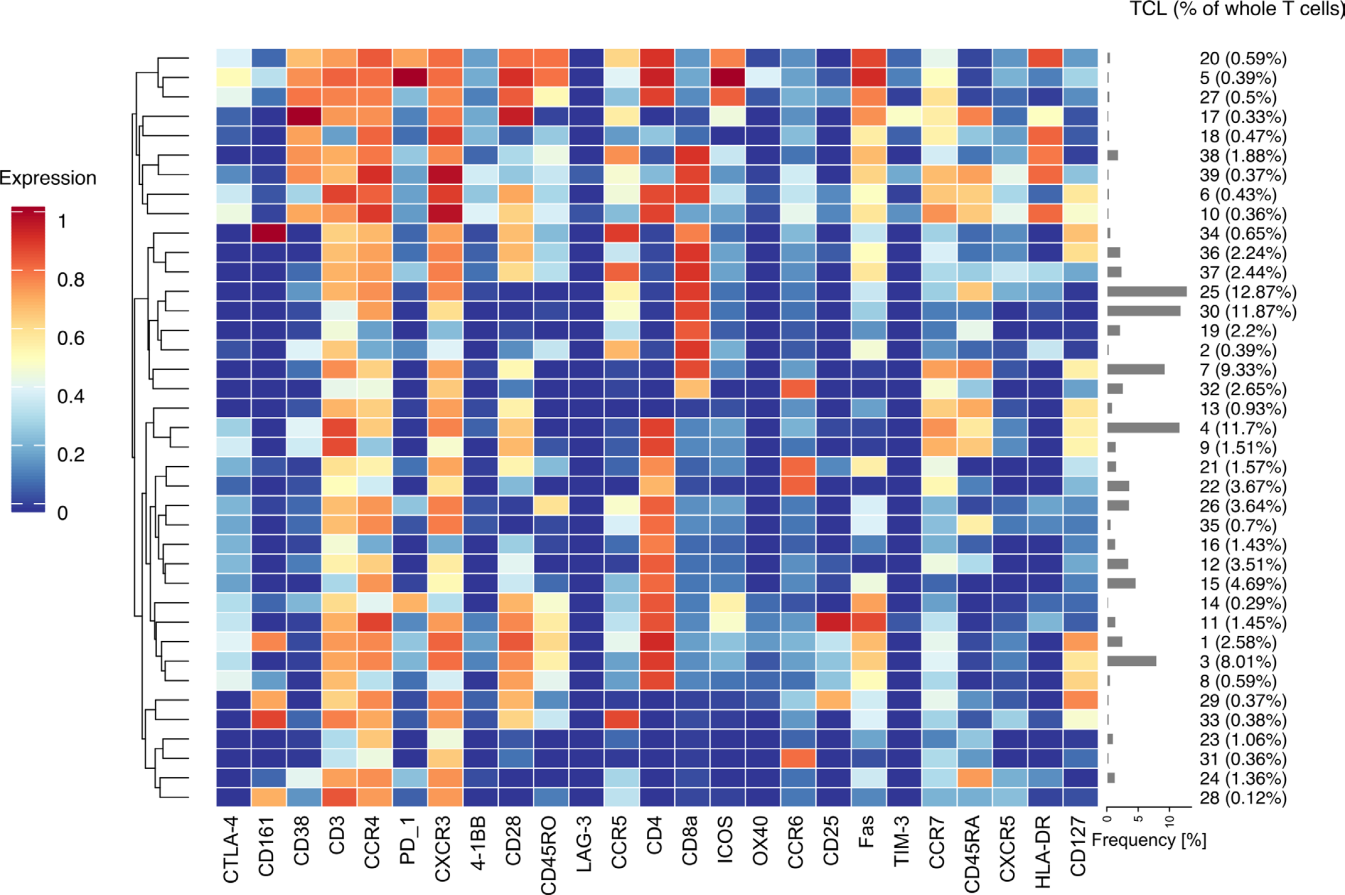
Immunophenotypic features of T-cell clusters (TCLs) CD3+ T-cell data from all 42 concatenated samples were clustered using the FlowSOM algorithm, which analyses high-dimensional cytometric data using self-organising maps, and 39 TCLs (cluster numbers 0–39) were identified. The expression of 25 surface markers for each TCL is shown in the heatmap. On the right, the percentage of each TCL (% of CD3+) is shown as a grey bar graph.

FlowSOM—a machine-learning algorithm—performs unsupervised clustering of high-dimensional cytometric data using self-organising maps. We performed T-cell clustering using this algorithm to identify the changes in peripheral blood T-cell phenotype before and after BEL treatment using changes in samples without BEL as controls. FlowSOM can reveal cell subsets that are difficult to identify by manual gating. Overall, 39 TCLs were identified. The expression levels of 25 T-cell surface markers for each TCL are shown in a heatmap (figure 2). Furthermore, the percentage of each TCL in each sample of CD3+ cells was analysed. The baseline values (% of CD3+) of the respective TCLs in both groups are shown in online supplemental table 4.

10.1136/lupus-2023-000976.supp11 Supplementary data

**Figure 3:**
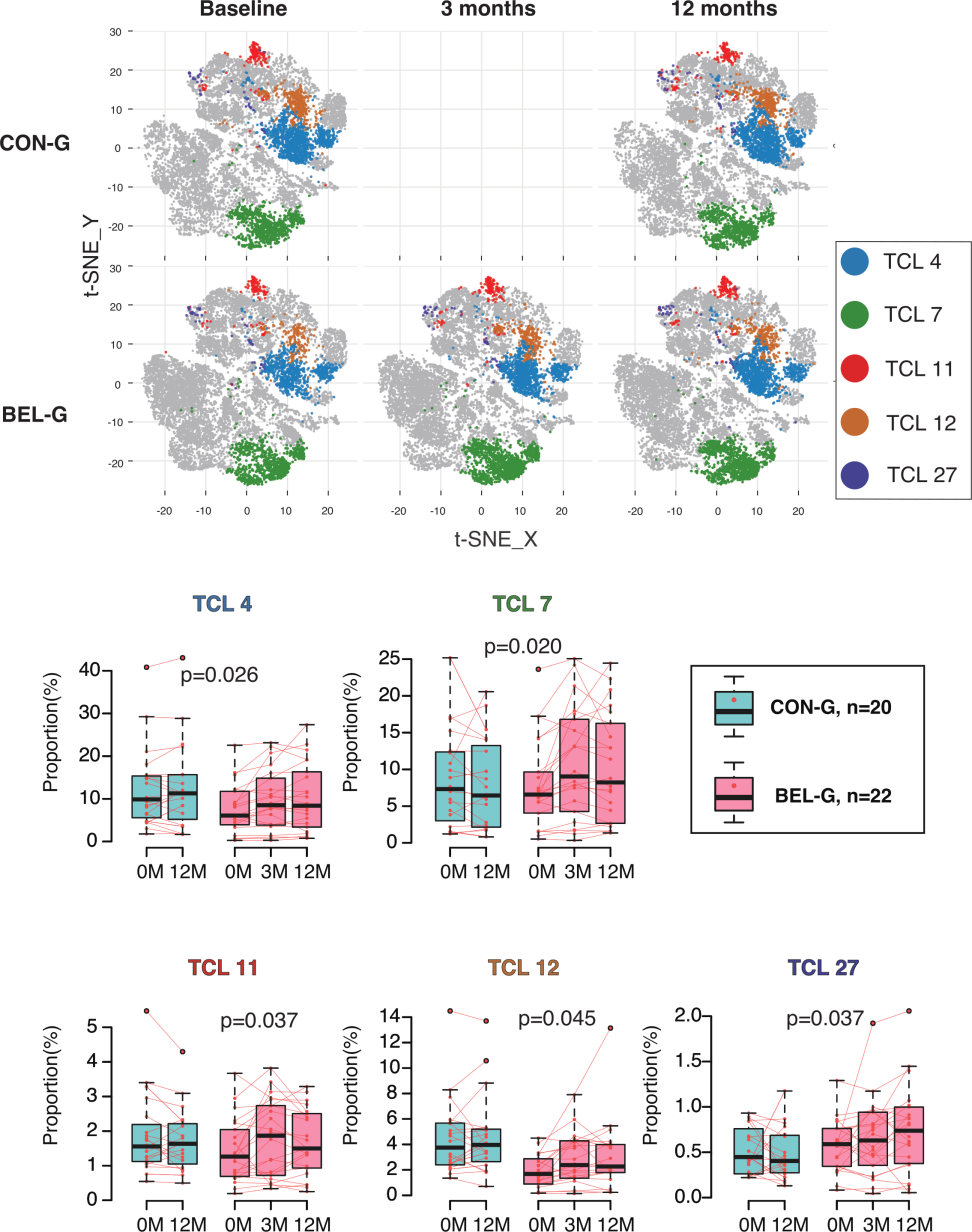
Belimumab (BEL) treatment-induced changes in T-cell clusters (TCLs). The five TCLs (TCL4, TCL7, TCL11, TCL12 and TCL2) that differed significantly between BEL group (BEL-G) and control group (CON-G) are shown in the t-distributed stochastic neighbour embedding (t-SNE) map by group and time (top panel). Whole CD3+ T cells are indicated by the grey dots. Changes in the proportions of the five TCLs (% of CD3+ T cells) are shown in a box-and-whisker plot (bottom graph). P values were calculated using a linear mixed-effects model.

High-dimensional concatenated data for 25 T-cell markers from 42 patients with SLE were visualised as two-dimensional plots using t-SNE. The expression of 25 cell surface markers was visualised as heatmaps on t-SNE maps (online supplemental figure 3). Then, t-SNE maps were separately displayed for BEL-G (0M, 3M and 12M) and CON-G (0M and 12M). The distribution of the 39 TCLs (TCL01–TCL39) on the t-SNE map was overlaid with the colour shown on the t-SNE map (online supplemental figure 4, figure 3). To compare groups of time-series data, TCLs (% of CD3+) showing significant associations with BEL treatment (BEL-TCL) were selected using a linear mixed-effects model. In this study, five distinct BEL-TCLs (TCL-04, 07, 11, 12 and 27) were identified (online supplemental table 5). Changes (% of CD3+) in these TCLs in BEL-G and CON-G are shown in figure 3.

10.1136/lupus-2023-000976.supp3 Supplementary data

10.1136/lupus-2023-000976.supp4 Supplementary data

10.1136/lupus-2023-000976.supp12 Supplementary data

TCL11, characterised by a Tregs phenotype (CD28+CXCR3+Fas+CD25high+CD127 CD4+ T), exhibited increased levels in the BEL-G (p=0.037). Similarly, TCL27, exhibiting a CM CD4+ T cells phenotype (CXCR3+CCR4+CD28+Fas+ICOS+activated CM CD4+ T), also showed elevated levels in the BEL-G (p=0.037). There was an increase in two naïve phenotype TCL: TCL04 (CD28+CTLA-4low+CXCR3+naïve CD4+ T, p=0.026) and TCL07 (CXCR3+CD28low+naïïve CD8+ T, p=0.02). TCL12 was a CD4+ TCL that expressed low levels of CXCR3 and CCR4 but was otherwise poorly characterised.

In a post hoc analysis, following our primary observations on T-cell subpopulations, we conducted a supplementary analysis to examine the relationship between TCL27 levels and complement titres at baseline for both BEL-G and CON-G groups (n=42). Employing Spearman’s correlation, a notable negative association was discerned between TCL27 levels and C3 complement titres, as illustrated in online supplemental figure 5. This relationship was statistically significant (p<0.05).

10.1136/lupus-2023-000976.supp5 Supplementary data

### Associations between BEL treatment and variations in Treg populations

**Figure 4:**
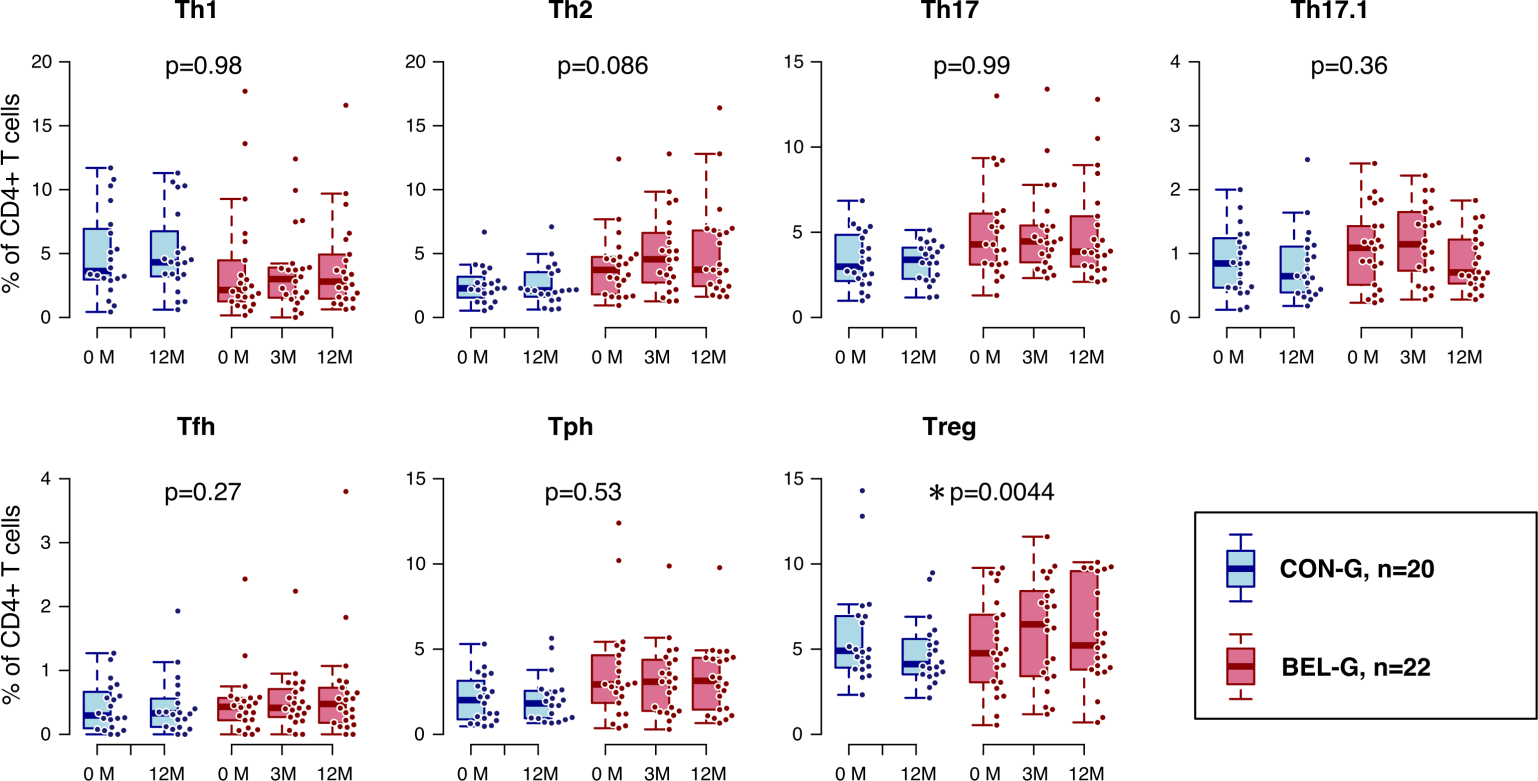
Helper T-cell changes induced by belimumab (BEL). The change in the percentage of each helper T-cell (% of CD4+ T cells) is shown using box-and-whisker plots. Both groups were compared, and p values were calculated using a linear mixed-effects model. P<0.05 was considered significant and indicated by an asterisk. BEL-G, BEL group; CON-G, control group.

**Figure 5:**
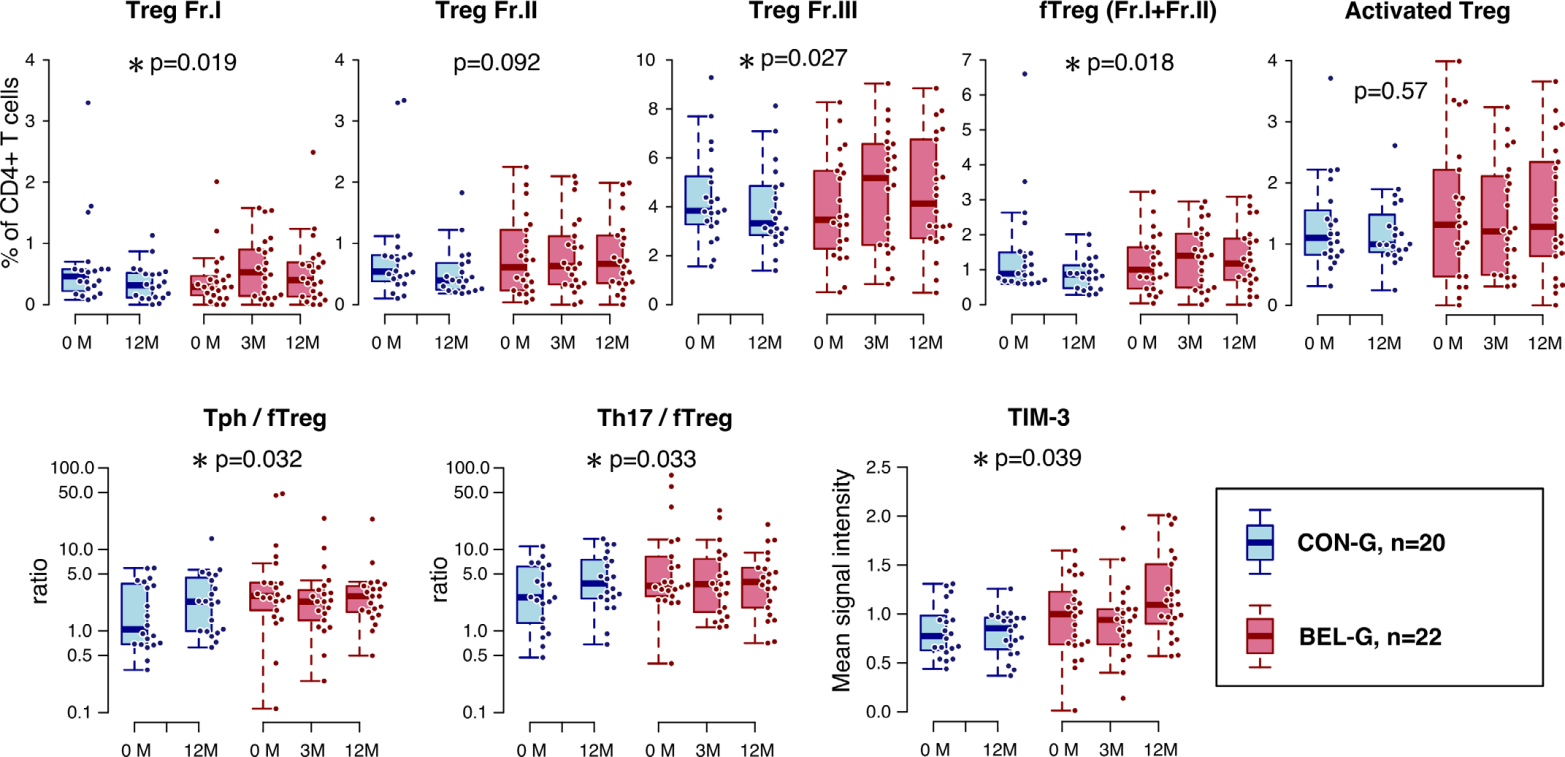
Effects of belimumab (BEL) on regulatory T cells (Tregs). Changes in Treg subsets (Treg Fr I, II, III, fTreg and activated Treg), Th/Treg balance (Th1/fTreg, Th2/fTreg, Th17/fTreg, Th17.1/fTreg, Tfh/fTreg and Tph/fTreg) and Treg cell surface molecule levels (CTLA-4, PD-1, 4-1BB, CD28, LAG-3, ICOS, OX-40, Fas, TIM-3 and HLA-DR) by BEL treatment were analysed using a linear mixed-effects model. Representative results are shown in the box-and-whisker plot. P<0.05 was considered significant and indicated by an asterisk. BEL-G, BEL group; CON-G, control group; CTLA-4, cytotoxic T-lymphocyte-associated antigen-4; HLA-DR, human leucocyte antigen-DR isotype; ICOS, inducible T-cell costimulator; LAG-3, lymphocyte-activation gene 3; PD-1, programmed death-1; TIM-3, T-cell immunoglobulin and mucin domain containing protein-3.

The Treg phenotype TCL11 exhibited increased levels in patients from the BEL-G. Consequently, our analysis shifted focus to Tregs and variations in T-cell subsets. Among CD4+ Th cells, Tregs were associated with higher levels in the BEL-G (p=0.0044) (online supplemental table 6, figure 4). We observed notable elevations in fraction I, fraction III and fTregs in the BEL-G (table 2, figure 5). Other Th cells did not show significant differences. Furthermore, we evaluated changes in the balance between Th cells and Treg. The ratio of each Th cell to fTreg was determined and analysed for changes by BEL; the ratio of Th17/fTreg (p=0.032) and Tph/fTreg (p=0.031) showed significant reductions in the BEL-G (table 2, figure 5). Various molecules are expressed on the surface of T cells depending on their status, including activation markers (HLA-DR and CD38), apoptosis-related molecules (Fas), costimulatory molecules (CD28, ICOS, OX40 and 4-1BB) and co-inhibitory molecules (CTLA-4, PD-1, LAG-3 and TIM-3). As mass cytometry can analyse the expression levels of many surface proteins at the single-cell level, we analysed changes in the expression levels (mean intensity value) of CTLA-4, PD-1, 4-1BB, CD28, LAG-3, ICOS, OX-40, Fas, TIM-3 and HLA-DR. We compared BEL-G and CON-G to evaluate the variations in surface molecules of Tregs associated with BEL treatment (online supplemental table 7, figure 5). Only the expression level of TIM-3 was increased in Tregs (p=0.039). Conventional manual gating analysis of CD4+ and CD8+ T cell (activated, CM, effector, EM and naïve) subsets did not identify subsets significantly affected by BEL (online supplemental table 8).

10.1136/lupus-2023-000976.supp13 Supplementary data

10.1136/lupus-2023-000976.supp14 Supplementary data

10.1136/lupus-2023-000976.supp15 Supplementary data

**Table 2:** Results of the analysis of changes in Treg subsets and Treg balance by BEL treatment using a linear mixed-effects model

	Estimate	95% CIs	SE	df	T value	P value
Treg subset (% CD4+ T cells)						
Treg Fr.I	0.4042	(0.0662 to 0.7423)	0.169	60.5816	2.3913	0.0199*
Treg Fr.II	0.3252	(−0.0554 to 0.7058)	0.1903	61.048	1.7085	0.0926
Treg Fr.III	1.0196	(0.1724 to 1.8668)	0.4362	61.3618	2.338	0.0227*
fTreg (Fr.I+Fr.II）	0.7294	(0.1475 to 1.3112)	0.2995	60.6479	2.435	0.0178*
Activated Treg (HLA-DR+CD38+)	0.1458	(−0.3698 to 0.6614)	0.258	62.3511	0.5652	0.5740
Th/fTreg (ratio)						
Th1/fTreg	−9.8917	(−21.8886 to 2.1052)	6.0077	65.3657	−1.6465	0.1045
Th2/fTreg	−5.1012	(−12.5676 to 2.3651)	3.7353	62.1743	−1.3657	0.1770
Th17/fTreg	−6.7868	(−12.9949 to −0.5787)	3.1038	60.2283	−2.1866	0.0327*
Th17.1/fTreg	−1.7032	(−3.5389 to 0.1325)	0.9188	63.712	−1.8537	0.0684
Tfh/fTreg	−0.4879	(−1.2567 to 0.2809)	0.3845	61.2111	−1.2689	0.2093
Tph/fTreg	−4.4775	(−8.5548 to −0.4002)	2.04	62.4936	−2.1948	0.0319*

Coefficients and p values were calculated using the linear mixed-effects model.

*Statistical significance level of p < 0.05.

Fr, fraction; fTreg, functional Treg (Fr.I+Fr.II); HLA-DR, human leucocyte antigen-DR isotype; Tfh, follicular helper T cells; Th, helper T cells; Tph, peripheral helper T cells; Treg, regulatory T cells.

### Post hoc correlation analysis between Treg, TCL11 and SLE activity markers

To further elucidate the potential associations between Treg, TCL11 and SLE activity markers, we conducted the following post hoc analyses:

Cross-sectional analysis at baseline (online supplemental figure 6): at the baseline, using data from a total of 42 patients, which included both BEL-treated patients (BEL-G, n=22) and non-BEL-treated controls (CON-G, n=20), we investigated correlations between the proportions of Tregs and TCL11 and SLE disease activity indicators: SLEDAI-2K, anti-dsDNA antibodies, CH50, C3, C4 and PGA. This analysis yielded no statistically significant correlations (online supplemental figure 6A). Moreover, when examining only the BEL-G cohort using data at 52 weeks post-treatment, we again found no significant correlations between the proportions of Tregs and TCL11 and the aforementioned disease activity markers (online supplemental figure 6B).

10.1136/lupus-2023-000976.supp6 Supplementary data

Longitudinal analysis over 52 weeks (online supplemental figure 7): we next calculated the change (Δ) from baseline to 52 weeks in the proportions of Tregs and TCL11 (ΔTreg, ΔTCL11), and assessed their correlations with changes in SLE activity markers (ΔSLEDAI-2K, Δanti-dsDNA antibodies, ΔCH50, ΔC3, ΔC4 and ΔPGA). Given our prior finding that Tregs and TCL11 were significantly increased in the BEL-G group compared with controls, we aimed to elucidate if these increases were linked to improvements (changes) in disease indicators.

10.1136/lupus-2023-000976.supp7 Supplementary data

In the non-BEL-treated CON-G, positive correlations were evident between ΔTreg and ΔTCL11 and changes in complement values (ΔCH50, ΔC3 and ΔC4). However, in the BEL-treated cohort, such correlations were not observed (online supplemental figure 7).

## Discussion

In the present study, we employed high-dimensional analysis, mass cytometry and machine-learning techniques to comprehensively analyse the changes in peripheral blood T cells induced by BEL treatment in patients with SLE. Five candidate BEL-TCLs (TCL04, 07, 11, 12 and 27) were identified. Pertaining to immune tolerance, we observed a significant increase in TCL11, which possesses a regulatory T-cell phenotype (CD4+CD25high+CD127−). Additionally, we observed an association between BEL treatment and an increased fraction of Tregs possessing regulatory function, as analysed by manual gating. The observed upsurge in Tregs might contribute to the stabilisation of SLE pathology, highlighting the therapeutic significance of BEL treatment. However, our analysis did not show a direct correlation between this upsurge and clinical indicators of disease activity, such as SLEDAI, dsDNA, C3, C4 and PGA. This suggests that the clinical therapeutic effect of BEL might be mediated through other immune-modulatory mechanisms, such as its main mode of action on B cell regulation, beyond the observed increase in Tregs. It is also possible that the influence of BEL on Tregs affects aspects of SLE pathology that are not directly captured by these commonly used clinical indices. Furthermore, our subsequent investigation revealed no direct correlation between changes in Treg and TCL11 and changes in complement levels. This suggests that there might be multiple contributing factors to the observed complement level improvements in the BEL-G. Conversely, in the CON-G, changes in Treg and TCL11 were directly linked to improvements in complement levels, underscoring the possibility of different immunomodulatory dynamics in the absence of BEL. BEL’s effects on Treg and TCL11 subsets in relation to SLE activity offer profound understanding into the drug’s multifaceted therapeutic impacts. Although its primary mode of action is through B-cell modulation, the observed secondary effects, especially on T-cell subsets, are pivotal. These observations suggest that, even post-BEL discontinuation, the altered levels of Treg and TCL11 could be crucial in maintaining SLE stability, highlighting the potential intrinsic role of these T-cell subsets, especially in inducing peripheral immune tolerance.

According to the results of the Th/fTreg ratio analysis, BEL was beneficial for balance between Th17/f Treg and Tph/f Treg. Another study reported an improved Th17/Treg balance,^47^ and our study supports their results. In this study, we further observed an association between BEL treatment and elevated levels of TIM-3 on Tregs. Notably, TIM-3 level was related to therapeutic responses to IFN-beta treatment and tocilizumab treatment in patients with multiple sclerosis and rheumatoid arthritis, respectively.^48^ Regarding TIM-3 expression in Tregs, several reports indicate that TIM-3 expression enhances the immunoregulatory capacity of Tregs, particularly in suppressing pathological behaviour in Th17.^49–51^ While the exact mechanism underlying the association between BEL treatment and the altered Th17/fTreg ratio remains unclear, we observed a coinciding rise in TIM-3 expression on Tregs and a favourable shift in the Th17/fTreg balance. However, further clarification of these mechanisms is warranted. This study demonstrated a novel beneficial effect of BEL on the Tph/fTreg balance in SLE. Accordingly, BEL administration in patients with SLE has been reported to improve levels of cytokines, including type 1 IFN,^52^ which contributes to Tph differentiation,^53^ promoting B-cell activation and inducing autoantibody production in SLE. The relative number of Tph is correlated with the disease activity of SLE.^54^ In this cohort, BEL significantly ameliorated the immunomodulatory abnormalities of SLE. This suggests that the alteration in the type 1 IFN level, as well as other yet unidentified factors affecting disease activity, could impact the Tph/fTreg balance. Thus, both direct and indirect effects of BEL on B cells and Tph/fTreg, respectively, may be involved in the suppression of B-cell hyperactivity by BEL.

Furthermore, in BEL-G, we observed a significant increase in two TCLs with naïve T-cell phenotype (TCL04 and TCL07). TCL04 was phenotypically closer to TCL09 among other TCLs (TCL6, TCL9 and TCL10) belonging to naïve CD4+ T cells and expressed slightly more CXCR3 and CCR4 than TCL09 but less CXCR3 and CCR4 than TCL06 and TCL10, which expressed them strongly. TCL10 was an activated naïve CD4T cell that expressed HLA-DR and CD38, which were not expressed by TCL04. TCL07 expressed lower levels of CXCR3 and CCR4 than other TCLs (TCL6 and TCL39) from the naïve CD8T cell group. TCL39 was an activated naïve CD8T cell that expressed HLA-DR and CD38; however, TCL07 did not express these. Moreover, TCL04 and TCL07 were characterised by the expression of Fas. Although features of naïve T cells involved in SLE pathogenesis, such as Fas expression on naïve T cells involved in lymphocytopenia,^55^ have been reported in patients with SLE, neither TCL04 nor TCL07 showed these features.

The search for pathological T-cell populations resistant to BEL treatment is critical for the development of novel treatment strategies. This machine-learning analysis identified an increase in TCL27, which is a phenotype of CM CD4+ T cells. TCL27 is characterised by a high expression of ICOS along with CD28 and CD38, which are highly expressed on activated T cells while not expressing PD-1. ICOS is highly expressed in peripheral blood T cells of patients with SLE and is involved in the dysregulation of T-cell activation and autoantibody production in SLE.^56^ Our post hoc analysis revealed a negative correlation between TCL27 and C3 complement titre in 42 baseline samples from both groups (online supplemental figure 5). This suggests that TCL27 may influence the immune dysregulations of SLE and could potentially serve as a new therapeutic target for immune remission under BEL treatment.

Several limitations of the present study should be noted. First, the analysed TCLs represent a subset classified based on the expression levels of 25 T-cell surface markers. Aspects related to T-cell function and differentiation were not considered in this study. TCL12, a CD4+ T-cell population with low expression of markers, was selected as BEL-TCL; however, its significance is not easily interpretable. Moreover, the definition of Th2 was not fully clarified as we did not analyse specific markers of Th2 apart from CCR4. Consequently, we refrained from drawing definitive conclusions about the effect of BEL on Th2 cells and TCL12. Second, relative apparent changes in specific TCLs may occur due to shifts in other TCL populations. Third, we did not analyse B cell dynamics, limiting our understanding of their relation to observed T cell changes. Lastly, we enrolled a relatively small number of patients compared with the number of T-cell subsets and TCLs analysed. The analysis was performed considering the actual clinical selection of BEL treatment. The backgrounds of the two patient groups were not matched. Therefore, statistically significant differences in individual comparisons were also selected and used as candidate BEL-TCLs. However, this exploratory study played a significant role in narrowing down the target cell population by identifying specific T-cell subsets impacted by BEL. Despite these limitations, our findings offer significant insights that pave the way for future investigations into the functional aspects of TCLs.

In conclusion, our findings emphasise the associations between BEL treatment and phenotypic changes in T cells of patients with SLE, highlighting the potential repercussions of targeted therapies on non-target immune cells. Specifically, we noted an association in the BEL-G with increased Tregs and shifts in the balances of Th17/Treg and Tph/Treg. Future research integrating molecular observations with clinical metrics could provide deeper insights into the impact of BEL treatment on Treg alterations and their clinical outcomes. Moreover, the observed TCL phenotype in CM CD4+ T cells post-BEL treatment opens a promising avenue for both achieving immunological remission in SLE and steering subsequent cellular functional studies.

10.1136/lupus-2023-000976.supp16 Supplementary data

## Data Availability

Data are available on reasonable request. All data relevant to the study are included in the article or uploaded as supplementary information.
