# Supplementary figures and images for: High-dimensional analysis of T-cell profiling variations following belimumab treatment in systemic lupus erythematosus
Source: Lupus Sci Med. 2023 Oct 6;10(2):e000976. doi: 10.1136/lupus-2023-000976 (PMC10565340; doi:10.1136/lupus-2023-000976)

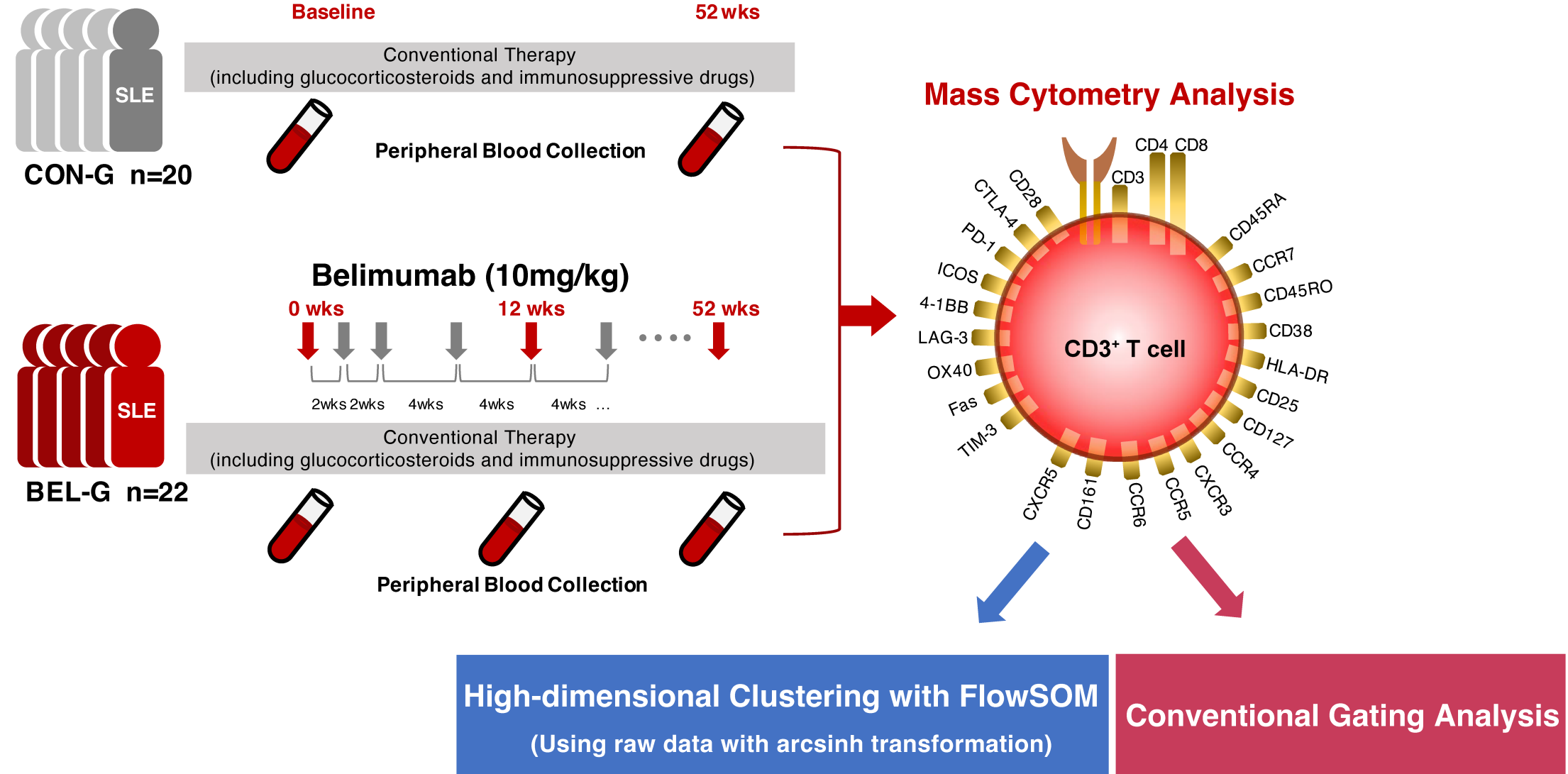

Supplement: Supplementary data [file lupus-2023-000976supp001.pdf]

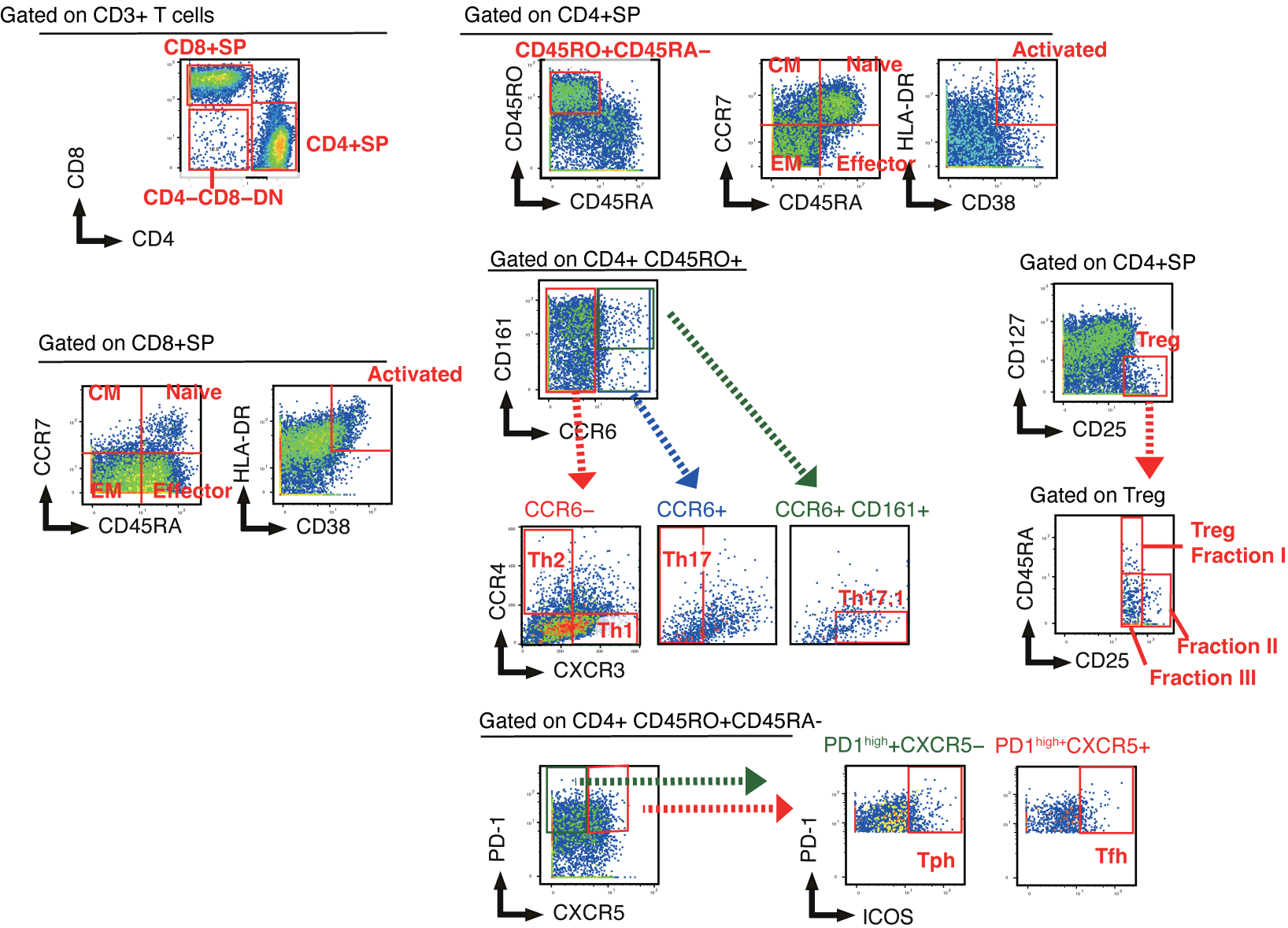

Supplement: Supplementary data [file lupus-2023-000976supp002.pdf]

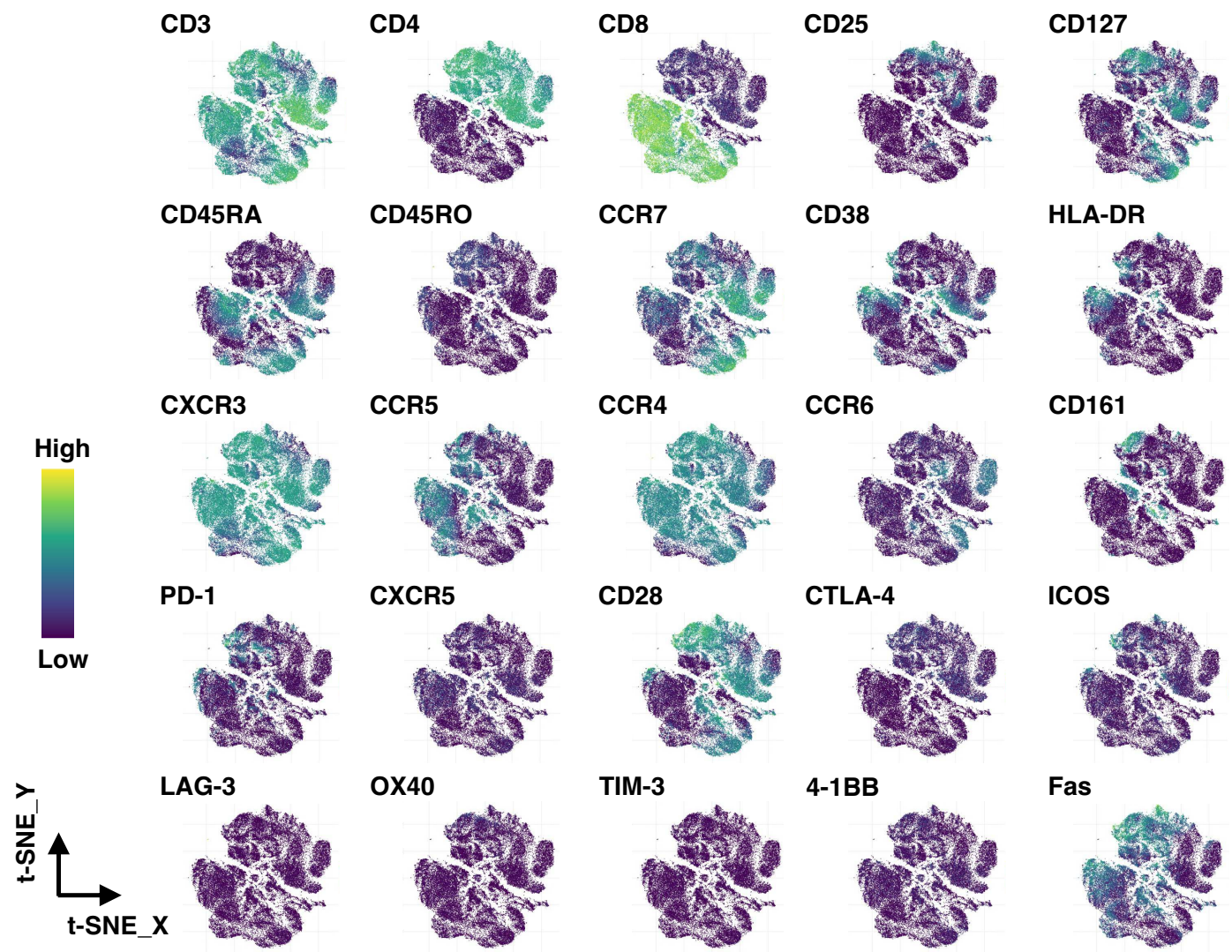

Supplement: Supplementary data [file lupus-2023-000976supp003.pdf]

|       |          |          |           |
|-------|----------|----------|-----------|
| CON-G | Baseline |          | 12 months |
| BEL-G | Baseline | 3 months | 12 months |

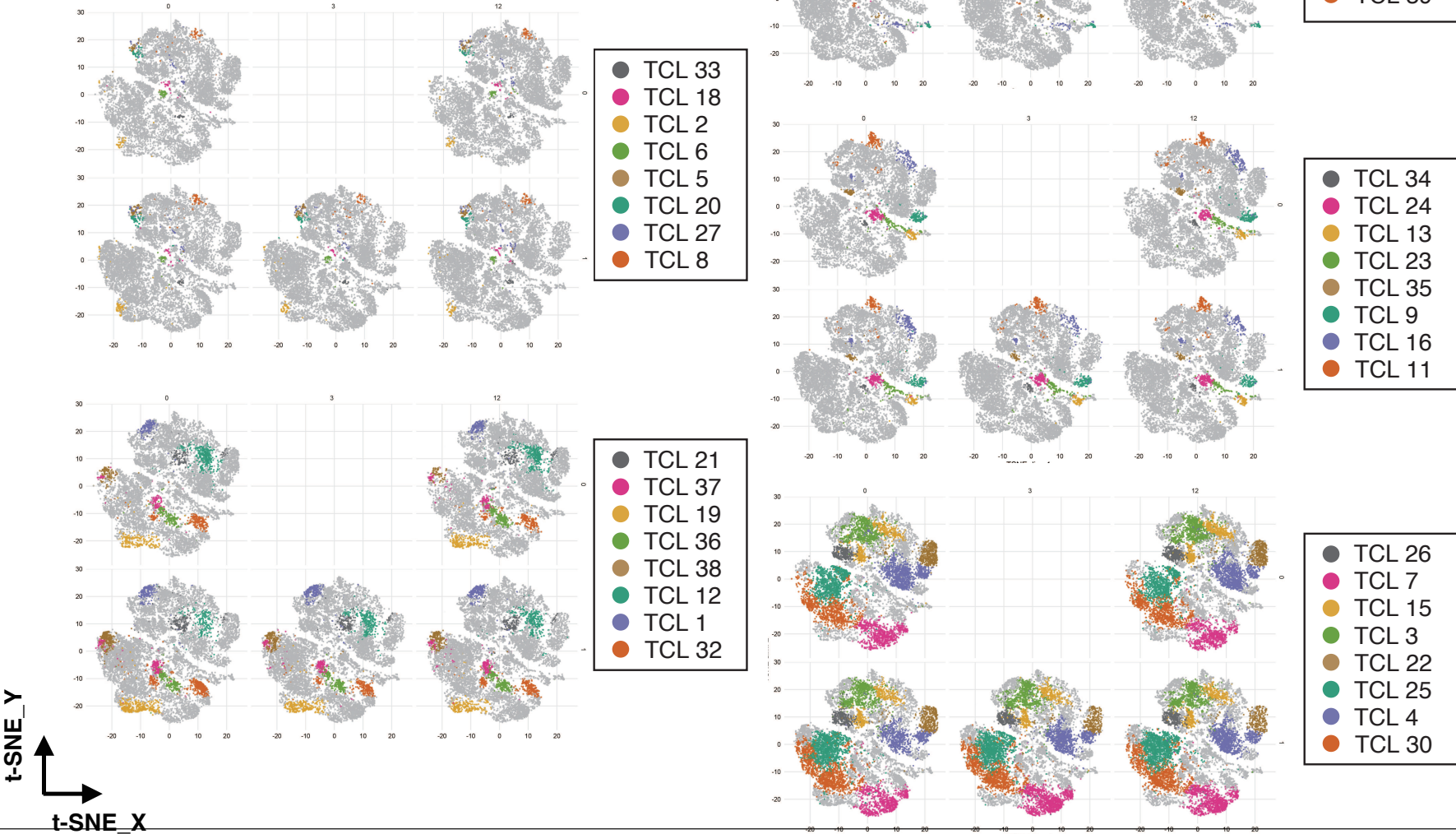

Supplement: Supplementary data [file lupus-2023-000976supp004.pdf]

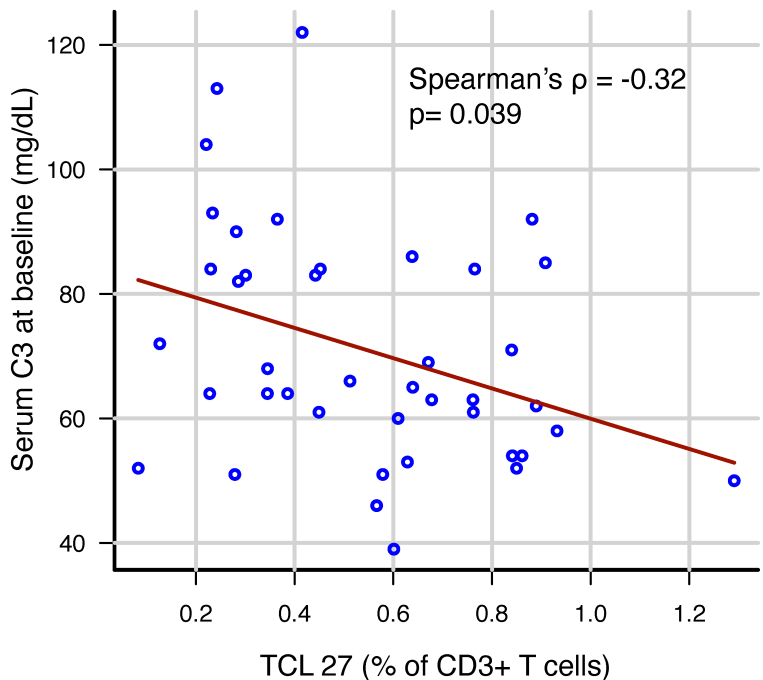

Supplement: Supplementary data [file lupus-2023-000976supp005.pdf]

**ΔTCL11**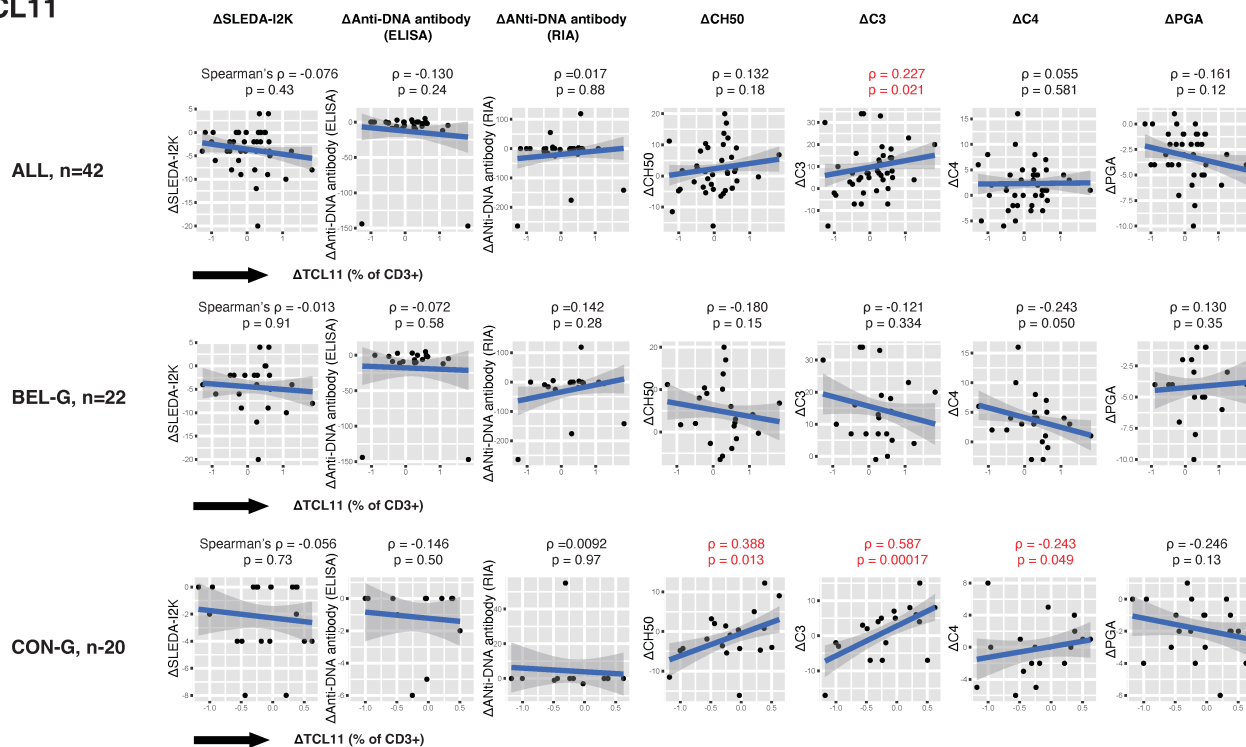**ΔTreg**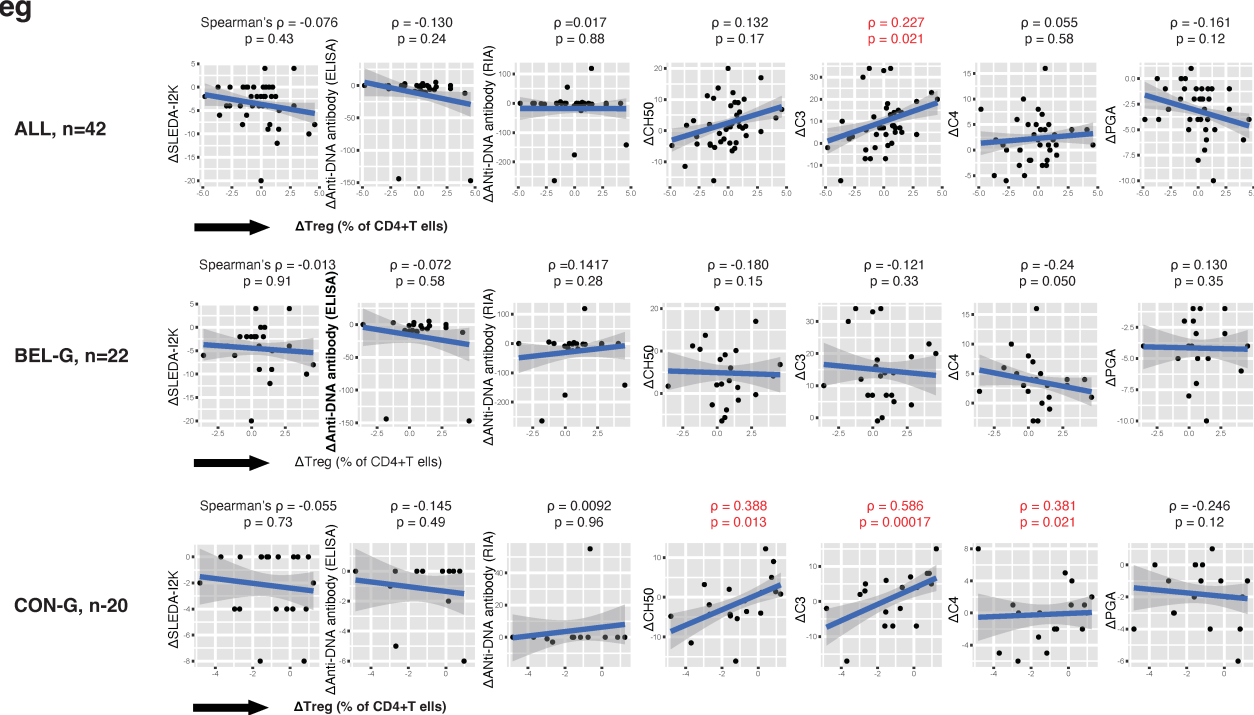

Supplement: Supplementary data [file lupus-2023-000976supp007.pdf]
