# Supplementary material for: High-dimensional analysis of T-cell profiling variations following belimumab treatment in systemic lupus erythematosus
Source: Lupus Sci Med. 2023 Oct 6;10(2):e000976. doi: 10.1136/lupus-2023-000976 (PMC10565340; doi:10.1136/lupus-2023-000976)
Supplement: Supplementary data [file lupus-2023-000976supp008.pdf]

Supplementary Table 1

List of metal-tagged antibodies used in mass cytometry experiments

CCR, C-C motif chemokine receptor; CXCR, C-X-C motif chemokine receptor; CTLA-4, cytotoxic T-lymphocyte-associated antigen-4; HLR-DR, human leukocyte antigen-DR isotype; ICOS, inducible T-cell costimulator; LAG-3, lymphocyte-activation gene 3; PD-1, programmed death-1; TIM-3, T-cell immunoglobulin and mucin domain containing protein-3

label	target	clone
170Er	CD3	UCHT1
145Nd	CD4	RPA-T4
146Nd	CD8a	RPA-T8
169Tm	CD45RA	HI100
165Ho	CD45RO	UCHL1
159Tb	CD197 (CCR7)	G043H7
167Er	CD38	HIT2
173Yb	HLA-DR	L243
149Sm	CD25 (IL-2R)	2A3
176Yb	CD127 (IL-7Ra)	A019D5
156Gd	CD183 (CXCR3)	G025H7
153Eu	CD194/CCR4	L291H4
144Nd	CD195 (CCR5)	NP-6G4
141Pr	CD196 (CCR6)	11A9
164Dy	CD161	HP-3G10
171Yb	CD185/CXCR5	51505
160Gd	CD28	CD28.2
161Dy	CD152 (CTLA-4)	14D3
155Gd	CD279 (PD-1)	EH12.2H7
148Nd	CD278/ICOS	C398.4A
158Gd	CD137/4-1BB	4B4-1
175Lu	CD223/LAG-3	11C3C65
150Nd	CD134 (OX40)	ACT35
152Sm	CD95/Fas	DX2
154Sm	TIM-3	F38-2E2
