# Supplementary material for: High-dimensional analysis of T-cell profiling variations following belimumab treatment in systemic lupus erythematosus
Source: Lupus Sci Med. 2023 Oct 6;10(2):e000976. doi: 10.1136/lupus-2023-000976 (PMC10565340; doi:10.1136/lupus-2023-000976)
Supplement: Supplementary data [file lupus-2023-000976supp009.pdf]

Supplementary Table 2

Results of a linear mixed-effects model analysis of changes in clinical indices of SLE and changes in immunoassays by BEL treatment

Coefficients and p-values were calculated using the linear mixed-effects model.

CI, confidence interval; df, degrees of freedom for the t-test; SE, standard error of the estimated effect in the model; SLEDAI-2K, SLE-Disease Activity Index 2000

	Estimate	SE	95%CI		df	t. value	p. value	
**disease activity**								
**SLEDAI-2K**	-0.1845	0.1015	-0.3864	0.0174	82.0000	-1.8176	0.0728	
**Physician Global Assessment (0 to 3 points)**	-0.0199	0.0048	-0.0294	-0.0105	74.0000	-4.1911	0.0001	*
**Blood, urine, and immunological tests**								
**Leukocytes**	-0.0504	0.0430	-0.1360	0.0352	82.0000	-1.1715	0.2448	
**Lymphocytes**	-0.0300	0.0241	-0.0780	0.0181	75.2639	-1.2424	0.2179	
**Hemoglobin**	0.0122	0.0266	-0.0408	0.0651	82.0000	0.4574	0.6486	
**Platelets**	-0.6593	1.2132	-3.0727	1.7541	82.0000	-0.5435	0.5883	
**Anti-double stranded DNA IgG antibody <ELISA>,**	-2.9936	1.6887	-6.3688	0.3817	62.3378	-1.7727	0.0812	
**Anti-DNA antibody <RIA>**	-1.4036	0.7386	-2.8795	0.0722	63.0804	-1.9005	0.0619	
**CH50**	0.4653	0.1526	0.1618	0.7689	82.0000	3.0493	0.0031	*
**C3**	1.1047	0.2340	0.6388	1.5706	78.2884	4.7204	0.0000	*
**C4**	0.2990	0.0913	0.1173	0.4808	78.3228	3.2750	0.0016	*
**Uine protein to creatinine ratio**	-0.0315	0.0203	-0.0719	0.0089	81.4325	-1.5514	0.1247	
**Drug**								
**Daily dose of prednisolone equivalent, mg**	-0.1769	0.0504	-0.2772	-0.0766	82.0000	-3.5076	0.0007	*
**Number of concomitant immunosuppressive and immunomodulatory agents**	0.0074	0.0088	-0.0101	0.0249	82.0000	0.8419	0.4023	
