# Supplementary material for: High-dimensional analysis of T-cell profiling variations following belimumab treatment in systemic lupus erythematosus
Source: Lupus Sci Med. 2023 Oct 6;10(2):e000976. doi: 10.1136/lupus-2023-000976 (PMC10565340; doi:10.1136/lupus-2023-000976)
Supplement: Supplementary data [file lupus-2023-000976supp010.pdf]

Supplementary Table 3

Peripheral blood T-cell phenotype at baseline

fTreg, functional Treg (Fr. I+ Fr. II); Tfh, follicular helper T cells; Th, helper T cells;

Tph, peripheral helper T cells; Treg, regulatory T cells

		CON-G, n=20	BEL-G, n=22	p.value	
T cell subset (%)					
	CD4-SP (in CD3+ T cells)	49.05 [41.30, 61.67]	42.15 [32.48, 48.53]	0.023	*
	activated (in CD4+ T cells)	4.27 (2.24)	6.82 (3.95)	0.015	*
	Central momory (in CD4+ T cells)	12.10 [9.92, 17.52]	19.65 [11.25, 24.22]	0.08	
	Effector (in CD4+ T cells)	6.56 [4.24, 11.53]	3.88 [2.23, 6.06]	0.02	*
	Effector memory (in CD4+ T cells)	57.35 [54.07, 68.10]	57.10 [51.55, 67.45]	0.87	
	Naive (in CD4+ T cells)	16.60 [12.51, 24.48]	15.25 [7.24, 18.80]	0.268	
	CD8-SP (in CD3+ T cells)	41.40 [25.60, 46.00]	48.45 [40.85, 54.10]	0.028	*
	activated (in CD8+ T cells)	7.23 [5.26, 15.67]	15.85 [8.69, 19.92]	0.048	*
	Central momory (in CD8+ T cells)	3.20 [2.23, 4.31]	3.94 [2.41, 5.22]	0.308	
	Effector (in CD8+ T cells)	34.25 [24.38, 43.02]	33.25 [20.40, 39.88]	0.58	
	Effector memory (in CD8+ T cells)	33.75 [24.63, 54.03]	44.90 [37.40, 54.82]	0.096	
	Naive (in CD8+ T cells)	17.55 [6.68, 30.40]	11.65 [5.20, 22.35]	0.29	
	CD4-CD8-double negative (in CD3+ Tcells)	5.33 [4.75, 8.93]	5.93 [3.73, 9.02]	0.782	
	CD4+CD8+double positive (in CD3+ Tcells)	0.66 [0.52, 0.85]	0.76 [0.50, 1.06]	0.481	
Th cell subsett (% of CD4+ Tcells)	Th1	3.63 [3.00, 6.76]	2.14 [1.33, 4.18]	0.059	
	Th2	2.28 [1.71, 3.03]	3.72 [1.98, 4.70]	0.027	*
	Th17	2.98 [2.21, 4.76]	4.28 [3.12, 5.98]	0.039	*
	Th17.1	0.84 [0.47, 1.21]	1.09 [0.58, 1.43]	0.279	
	Tfh	0.30 [0.10, 0.63]	0.43 [0.22, 0.56]	0.87	
	Tph	2.01 [0.91, 2.99]	2.93 [1.94, 4.53]	0.066	
	Treg	4.90 [3.95, 6.93]	4.76 [3.06, 6.97]	0.554	
	Treg Fr. I	0.46 [0.24, 0.58]	0.29 [0.16, 0.45]	0.158	
	Treg Fr. II	0.54 [0.40, 0.80]	0.61 [0.25, 1.18]	0.9	
	Treg Fr.III	3.84 [3.32, 5.12]	3.46 [2.36, 5.45]	0.406	
	functional Treg (Fr. I + Fr. II)	0.88 [0.68, 1.48]	1.00 [0.53, 1.64]	0.791	
Th/ functional Treg (ratio)	Th1/fTreg	0.70 [0.48, 1.36]	0.60 [0.22, 1.14]	0.351	
	Th2/fTreg	0.40 [0.29, 0.60]	0.68 [0.60, 0.90]	0.004	*
	Th17/fTreg	0.63 [0.38, 0.78]	0.89 [0.74, 1.92]	0.009	*
	Th17.1/fTreg	0.13 [0.10, 0.21]	0.21 [0.12, 0.39]	0.074	
	Tfh/fTreg	0.07 [0.04, 0.11]	0.08 [0.06, 0.13]	0.351	
	Tph/fTreg	0.31 [0.18, 0.54]	0.59 [0.48, 1.07]	0.008	*
