# Supplementary material for: High-dimensional analysis of T-cell profiling variations following belimumab treatment in systemic lupus erythematosus
Source: Lupus Sci Med. 2023 Oct 6;10(2):e000976. doi: 10.1136/lupus-2023-000976 (PMC10565340; doi:10.1136/lupus-2023-000976)
Supplement: Supplementary data [file lupus-2023-000976supp011.pdf]

Supplementary Table 4

T-cell clusters at baseline

The percentages (% of CD3+ T cells) of each peripheral blood T-cell cluster (TCL, median [first quartile and third quartile]) in BEL-G and CON-G are shown.

TCL	CON-G, n=20	BEL-G, n=22	p.value	
1	1.28 [1.02, 2.30]	2.09 [0.97, 2.77]	0.497	
2	0.18 [0.01, 0.30]	0.49 [0.20, 0.87]	0.016	*
3	7.12 [5.31, 11.53]	5.65 [4.70, 8.16]	0.29	
4	9.86 [5.97, 15.21]	6.07 [4.06, 11.11]	0.07	
5	0.21 [0.09, 0.55]	0.49 [0.28, 0.65]	0.037	*
6	0.41 [0.31, 0.52]	0.26 [0.17, 0.43]	0.074	
7	7.31 [3.42, 12.31]	6.56 [4.43, 9.46]	0.821	
8	0.17 [0.09, 0.61]	0.22 [0.12, 0.77]	0.513	
9	0.37 [0.18, 1.46]	0.49 [0.18, 0.98]	0.92	
10	0.28 [0.20, 0.42]	0.20 [0.11, 0.42]	0.268	
11	1.56 [1.14, 2.07]	1.26 [0.70, 1.99]	0.151	
12	3.75 [2.40, 5.65]	1.68 [0.90, 2.82]	0.000227	*
13	0.71 [0.44, 1.15]	0.67 [0.28, 1.03]	0.529	
14	0.20 [0.07, 0.41]	0.23 [0.12, 0.45]	0.45	
15	4.79 [3.32, 6.83]	3.67 [2.80, 5.00]	0.208	
16	0.84 [0.59, 1.47]	0.54 [0.36, 3.17]	0.199	
17	0.37 [0.23, 0.59]	0.33 [0.12, 0.66]	0.614	
18	0.44 [0.35, 0.63]	0.31 [0.18, 0.46]	0.047	*
19	1.07 [0.43, 2.01]	0.83 [0.50, 4.80]	0.98	
20	0.52 [0.32, 0.75]	0.57 [0.33, 0.89]	0.58	
21	1.40 [0.92, 1.94]	1.74 [1.39, 2.44]	0.144	
22	5.27 [3.42, 7.43]	3.11 [1.59, 5.03]	0.074	
23	1.38 [0.92, 1.64]	1.09 [0.61, 1.67]	0.326	
24	1.31 [0.70, 2.12]	1.36 [0.53, 1.86]	0.94	
25	10.19 [5.92, 14.08]	11.53 [7.02, 15.65]	0.351	
26	3.23 [1.43, 5.98]	2.08 [0.88, 3.90]	0.435	
27	0.45 [0.27, 0.72]	0.59 [0.34, 0.76]	0.687	
28	0.07 [0.02, 0.13]	0.02 [0.00, 0.12]	0.253	
29	0.13 [0.07, 0.42]	0.17 [0.09, 0.35]	0.753	
30	10.18 [5.59, 14.32]	13.93 [8.66, 17.29]	0.074	
31	0.45 [0.20, 0.85]	0.39 [0.20, 0.59]	0.513	
32	2.13 [1.06, 4.27]	3.09 [1.79, 5.27]	0.19	
33	0.11 [0.04, 0.44]	0.07 [0.03, 0.21]	0.687	
34	0.24 [0.11, 0.54]	0.15 [0.05, 0.42]	0.29	
35	0.19 [0.03, 0.89]	0.13 [0.04, 0.24]	0.65	
36	1.29 [0.66, 2.67]	1.16 [0.84, 3.03]	0.821	
37	0.58 [0.43, 2.03]	1.41 [0.79, 2.50]	0.078	
38	0.63 [0.35, 1.81]	1.74 [0.77, 3.69]	0.013	*
39	0.25 [0.18, 0.37]	0.28 [0.19, 0.41]	0.782	
