# Supplementary material for: High-dimensional analysis of T-cell profiling variations following belimumab treatment in systemic lupus erythematosus
Source: Lupus Sci Med. 2023 Oct 6;10(2):e000976. doi: 10.1136/lupus-2023-000976 (PMC10565340; doi:10.1136/lupus-2023-000976)
Supplement: Supplementary data [file lupus-2023-000976supp012.pdf]

Supplementary Table 5

Results of the analysis of changes in T-cell clusters by BEL treatment using a linear mixed-effects model

Coefficients and p-values were calculated using the linear mixed-effects model.

CI, confidence interval; df, degrees of freedom for the t-test; SE, standard error of the estimated effect in the model

TCL	Coefficient (95%CI)	Std.Error	df	t value	p value
1	0.238 (-0.437, 0.913)	0.347	61.425	0.684	*0.496*
2	0.241 (-0.711, 0.228)	0.242	61.267	-0.998	*0.322*
3	0.362 (-1.219, 1.942)	0.814	61.119	0.444	*0.658*
4	2.29 (0.334, 4.245)	1.007	60.810	2.275	*0.026* *
5	0.029 (-0.149, 0.207)	0.092	61.553	0.315	*0.754*
6	-0.032 (-0.338, 0.275)	0.158	62.966	-0.203	*0.840*
7	2.978 (0.561, 5.395)	1.244	61.142	2.394	*0.020* *
8	-0.287 (-0.728, 0.154)	0.227	62.159	-1.264	*0.211*
9	-0.435 (-1.105, 0.234)	0.345	60.781	-1.264	*0.211*
10	-0.194 (-0.514, 0.126)	0.165	64.144	-1.176	*0.244*
11	0.404 (0.035, 0.772)	0.190	60.918	2.130	*0.037* *
12	1.065 (0.053, 2.077)	0.521	60.285	2.045	*0.045* *
13	0.155 (-0.061, 0.37)	0.111	61.049	1.395	*0.168*
14	-0.127 (-0.504, 0.25)	0.194	62.712	-0.652	*0.517*
15	-0.137 (-0.802, 0.528)	0.342	60.730	-0.400	*0.691*
16	-0.673 (-1.865, 0.52)	0.614	61.969	-1.096	*0.277*
17	-0.079 (-0.259, 0.102)	0.093	60.084	-0.845	*0.401*
18	-0.012 (-0.187, 0.163)	0.090	61.740	-0.135	*0.893*
19	-1.619 (-4.107, 0.869)	1.281	62.760	-1.264	*0.211*
20	-0.065 (-0.329, 0.199)	0.136	62.380	-0.479	*0.633*
21	-0.114 (-0.799, 0.571)	0.353	61.546	-0.323	*0.748*
22	0.313 (-1.407, 2.033)	0.885	59.672	0.354	*0.725*
23	0.008 (-0.574, 0.59)	0.300	63.602	0.027	*0.979*
24	0.372 (-0.44, 1.184)	0.418	62.419	0.889	*0.377*
25	1.214 (-2.78, 5.207)	2.057	62.143	0.590	*0.557*
26	-0.818 (-1.749, 0.113)	0.479	60.960	-1.707	*0.093*
27	0.222 (0.02, 0.425)	0.104	62.930	2.130	*0.037* *
28	0.003 (-0.08, 0.085)	0.042	60.559	0.065	*0.948*
29	0.073 (-0.07, 0.215)	0.073	61.102	0.992	*0.325*
30	-3.602 (-7.272, 0.069)	1.890	60.609	-1.906	*0.061*
31	0.205 (-0.227, 0.637)	0.222	55.870	0.923	*0.360*
32	-0.666 (-2.016, 0.685)	0.696	61.983	-0.957	*0.342*
33	-0.105 (-0.23, 0.02)	0.064	61.225	-1.626	*0.109*
34	0.012 (-0.278, 0.302)	0.149	61.809	0.080	*0.936*
35	-0.103 (-0.31, 0.105)	0.107	60.923	-0.963	*0.339*
36	0.009 (-0.709, 0.727)	0.369	60.771	0.025	*0.981*
37	-0.183 (-0.894, 0.529)	0.366	61.594	-0.499	*0.620*
38	-0.194 (-1.325, 0.937)	0.582	63.266	-0.333	*0.740*
39	-0.215 (-0.659, 0.23)	0.229	65.923	-0.937	*0.352*
