# Supplementary material for: High-dimensional analysis of T-cell profiling variations following belimumab treatment in systemic lupus erythematosus
Source: Lupus Sci Med. 2023 Oct 6;10(2):e000976. doi: 10.1136/lupus-2023-000976 (PMC10565340; doi:10.1136/lupus-2023-000976)
Supplement: Supplementary data [file lupus-2023-000976supp013.pdf]

Supplementary Table 6

Results of the analysis of changes in helper T-cell subsets by BEL treatment using a linear mixed-effects model

Coefficients and p-values were calculated using the linear mixed-effects model.

CI, confidence interval; df, degrees of freedom for the t-test; SE, standard error of the estimated effect in the model

Th cell subset (% CD4+ T cells)	Estimate	confidence intervals	Std. Error	df	t value	p.value	
Th1	0.0077	(-1.1413,1.1567)	0.5746	61.1604	0.0134	0.9894	
Th2	1.1565	(-0.169,2.4821)	0.6633	62.6762	1.7438	0.0861	
Th17	0.0035	(-1.1037,1.1107)	0.554	62.4751	0.0063	0.9950	
Th17.1	-0.1566	(-0.4956,0.1824)	0.1695	60.6501	-0.9238	0.3593	
Tfh	0.1537	(-0.1242,0.4316)	0.139	62.3255	1.1053	0.2733	
Tph	-0.2976	(-1.245,0.6498)	0.474	62.1815	-0.628	0.5323	
Treg	1.749	(0.5654,2.9326)	0.592	61.2857	2.9545	0.0044	*
