# Supplementary material for: High-dimensional analysis of T-cell profiling variations following belimumab treatment in systemic lupus erythematosus
Source: Lupus Sci Med. 2023 Oct 6;10(2):e000976. doi: 10.1136/lupus-2023-000976 (PMC10565340; doi:10.1136/lupus-2023-000976)
Supplement: Supplementary data [file lupus-2023-000976supp014.pdf]

Supplementary Table 7

Results of the analysis of changes in expression levels of Treg cell surface molecules by BEL treatment using linear mixed-effects models

Coefficients and p-values were calculated using the linear mixed-effects model.

CI, confidence interval; df, degrees of freedom for the t-test; SE, standard error of the estimated effect on the model

Treg surface molecules（M.I.）	Estimate	confidence intervals	Std. Error	df	t value	p.value	
CTLA-4	-0.4179	(-0.9984,0.1626)	0.2907	65.3151	-1.4377	0.1553	
PD-1	1.3098	(-0.667,3.2866)	0.9897	64.701	1.3234	0.1904	
4-1BB	0.026	(-0.2194,0.2713)	0.1228	63.2441	0.2114	0.8333	
CD28	1.5891	(-5.0786,8.2568)	3.3332	59.8404	0.4768	0.6353	
LAG-3	0.0264	(-0.1584,0.2111)	0.0926	69.3131	0.2846	0.7768	
ICOS	-2.8088	(-6.5662,0.9487)	1.8812	64.5517	-1.4931	0.1403	
OX-40	-0.003	(-0.4594,0.4535)	0.2283	61.7166	-0.0129	0.9897	
Fas	-2.3595	(-12.9874,8.2684)	5.3198	63.9131	-0.4435	0.6589	
TIM-3	0.2422	(0.0126,0.4718)	0.1149	63.4554	2.1076	0.0390	*
HLA-DR	1.245	(-8.2516,10.7415)	4.7542	64.3951	0.2619	0.7943	
