# Supplementary material for: High-dimensional analysis of T-cell profiling variations following belimumab treatment in systemic lupus erythematosus
Source: Lupus Sci Med. 2023 Oct 6;10(2):e000976. doi: 10.1136/lupus-2023-000976 (PMC10565340; doi:10.1136/lupus-2023-000976)
Supplement: Supplementary data [file lupus-2023-000976supp015.pdf]

Supplementary Table 8

Analysis of changes in CD4+ T-cell subsets, CD8+ T-cell subsets, and CD4-CD8-double-negative T cells by BEL treatment using linear mixed-effects models

CI, confidence interval; df, degrees of freedom for the t-test; SE, standard error of the estimated effect on the model; SP, single positive

T cell subset (% of CD3+ Tcells)	Estimate	confidence intervals	Std. Error	df	t value	p.value
CD4-SP	1.4064	(-3.5778,6.3905)	2.492	60.4024	0.5643	0.5746
Activated CD4-SP	0.0891	(-0.6385,0.8168)	0.364	61.7885	0.2448	0.8074
Central memory CD4-SP	0.3456	(-1.5041,2.1953)	0.9251	61.3787	0.3735	0.7100
Effector CD4-SP	-0.2289	(-2.1049,1.6472)	0.9378	59.8748	-0.244	0.8080
Effector memory CD4-SP	-0.0856	(-3.2152,3.0441)	1.5651	60.9105	-0.0547	0.9566
Naïve CD4-SP	1.3688	(-0.6395,3.3772)	1.0043	60.7301	1.363	0.1779
CD8-SP	-1.8971	(-6.9582,3.164)	2.5305	60.3506	-0.7497	0.4563
Activated CD8-SP	-0.3473	(-2.937,2.2423)	1.2959	62.9958	-0.268	0.7896
Central memory CD8-SP	-0.0944	(-0.6376,0.4489)	0.2718	62.192	-0.3473	0.7296
Effector CD8-SP	0.2932	(-2.3758,2.9622)	1.3348	61.1196	0.2197	0.8269
Effector memory CD8-SP	-4.2754	(-9.0904,0.5396)	2.4076	60.5224	-1.7758	0.0808
Naïve CD8-SP	2.1817	(-0.0072,4.3706)	1.0947	61.1835	1.9929	0.0507
CD4-CD8- double negative T cells	0.5612	(-1.0128,2.1353)	0.7873	61.6289	0.7128	0.4786
