# Supplementary material for: High-dimensional analysis of T-cell profiling variations following belimumab treatment in systemic lupus erythematosus
Source: Lupus Sci Med. 2023 Oct 6;10(2):e000976. doi: 10.1136/lupus-2023-000976 (PMC10565340; doi:10.1136/lupus-2023-000976)

A. Correlation of TCL11 and Treg with Clinical Parameters at Baseline in 42 Cases of SLE Patients

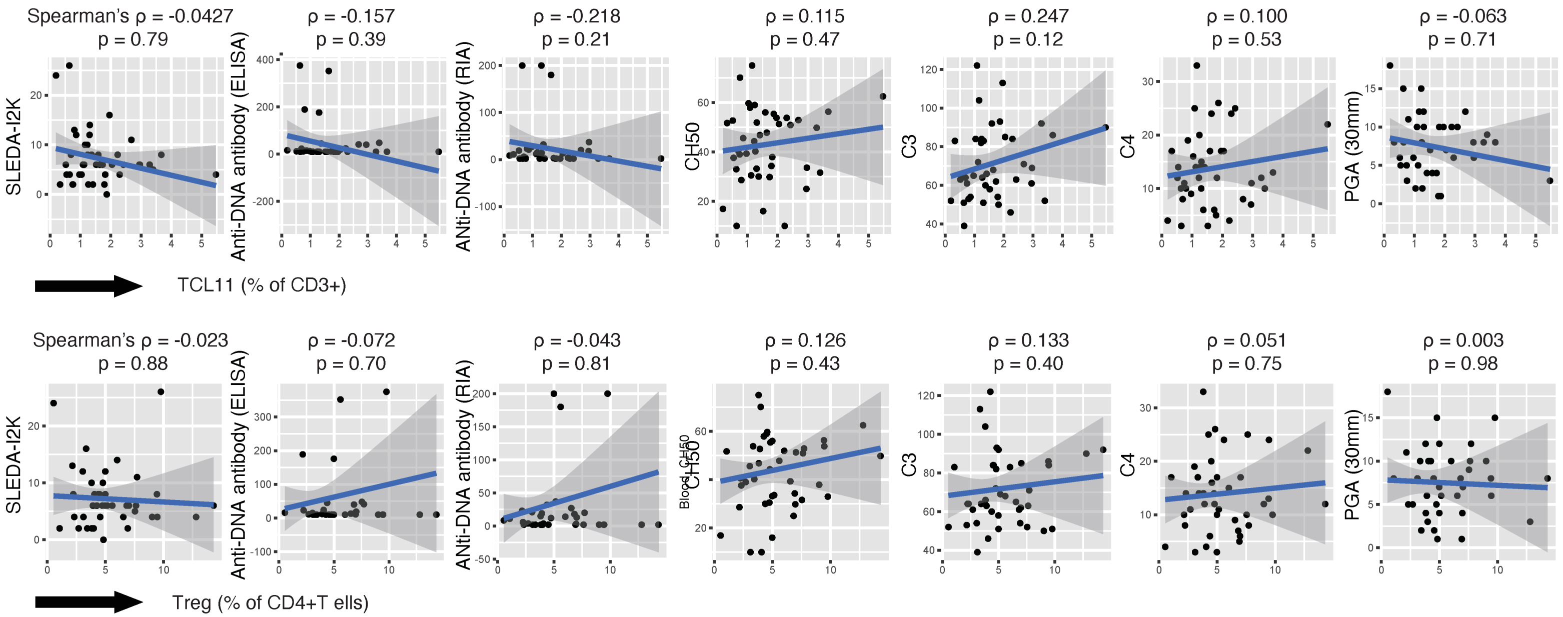

B. Correlation of TCL11 and Treg with Clinical Parameters at 52 Weeks After BELIMUMAB Treatment in the BEL-G

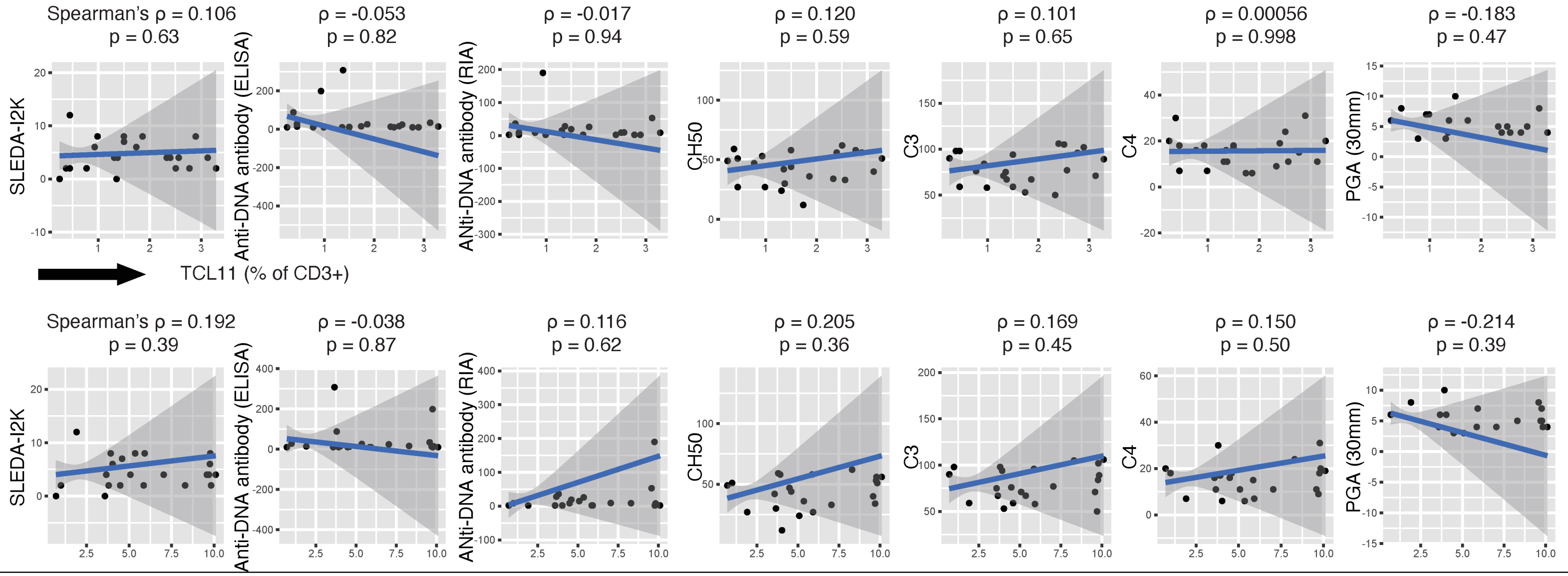

Supplement: Supplementary data [file lupus-2023-000976supp006.pdf]
