# Supplementary material for: High-dimensional analysis of T-cell profiling variations following belimumab treatment in systemic lupus erythematosus
Source: Lupus Sci Med. 2023 Oct 6;10(2):e000976. doi: 10.1136/lupus-2023-000976 (PMC10565340; doi:10.1136/lupus-2023-000976)
Supplement: Supplementary data [file lupus-2023-000976supp016.pdf]

Supplementary FIgure Legend

Supplementary Figure 1

SLE Treatment Timeline and Mass Cytometry Data Analysis Workflow

The diagram delineates the treatment and sampling timeline for Systemic Lupus Erythematosus (SLE) patients under Belimumab (BEL-G) and the control group (CON-G). Blood samples for the BEL-G cohort were taken at 0 weeks, 12 weeks, and 52 weeks, while for the CON-G, they were drawn at baseline and then at 52 weeks. From these samples, T-cell populations were analyzed, with all target surface antigens for our Mass Cytometry analysis illustrated on the depicted T-cell. Analysis subsequently diverges into two methods: 1) High-dimensional Clustering with FlowSOM using raw data with arcsinh transformation, and 2) Traditional Conventional Gating Analysis. This bifurcated approach offers both a cutting-edge, detailed view of the cellular profile and grounds itself in established cytometric techniques, assuring thorough data interpretation.

Supplementary Figure 2

Gating strategy for the identification of T-cell subsets

The cytometric plots showing a gating strategy that identifies Th1, Th2, Th17, Th17.1, peripheral helper T (Tph), follicular helper T (Tfh), regulatory T (Treg) cells in the peripheral blood, and three fractions of Tregs (Fr I, II, and III) as well as central memory (CM), EM, effector, naive, and activated T cells.

BMJ Publishing Group Limited (BMJ) disclaims all liability and responsibility arising from any reliance placed on this supplemental material which has been supplied by the author(s)

Supplementary Figure 3

Expression levels of the 25 T-cell markers on the t-SNE map

Cytometry data for CD3+ T-cell gating of all samples (n=42) were collected. To visualize the high-dimensional concatenated data, dimensionality reduction was performed using the t-distributed stochastic neighbor embedding (t-SNE) technique (x-axis, t-SNE-x; y-axis, t-SNE-y). The expression levels of each of the 25 markers are shown as a heat map on the t-SNE map.

Supplementary Figure 4

Display of all T-cell clusters on the t-SNE map

The 39 T-cell clusters are shown in the t-SNE map by group and time. All 39 T-cell clusters were divided into five parts to avoid overlapping on the t-SNE map, and each T-cell cluster is shown in the color indicated. Whole CD3+ T cells are indicated by gray dots.

Supplementary Figure 5

Correlation analysis of TCL27 and serum C3 levels at baseline

The scatter plot shows the serum C3 level and the percentage of TCL27 (% of CD3+ T cells) using all 42 samples (without BEL) at baseline, and the regression line (red line), Spearman’s rank correlation coefficient rho (ρ), and p-value are shown in the plot.

Supplementary Figure 6

Correlation of TCL11 and Treg with Clinical Parameters

(A) Baseline for 42 SLE Patients, combining data from both Control Group (CON-G) and BELIMUMAB Treatment Group (BEL-G).

(B) 52 Weeks Post-BELIMUMAB Treatment exclusively for the BEL-G Group.

For both (A) and (B), scatter plots feature a blue regression line with Spearman's ρ values and associated p-values annotated above. None of the correlations reached statistical significance.

Supplementary Figure 7

Relationship of ΔTreg and ΔTCL11 with SLE Markers Over a Year

Longitudinal correlations over 52 weeks between changes (Δ) in Treg and TCL11 proportions and changes in SLE activity markers, visualized using scatter plots. Analyses were conducted using Spearman's rank correlation coefficient rho (ρ), and both ρ and p-values are displayed on the graphs. Significance was set at $p < 0.05$. For the non-Belimumab-treated CON-G, significant positive correlations were noted between ΔTreg and ΔTCL11 and changes in complement values (ΔCH50, ΔC3, and ΔC4). Conversely, in the BEL-G cohort, these correlations were absent.
